# agReg-SNPdb-Plants: A Database of Regulatory SNPs for Agricultural Plant Species

**DOI:** 10.3390/biology11050684

**Published:** 2022-04-29

**Authors:** Selina Klees, Felix Heinrich, Armin Otto Schmitt, Mehmet Gültas

**Affiliations:** 1 Breeding Informatics Group, Department of Animal Sciences, Georg-August University, Margarethe von Wrangell-Weg 7, 37075 Göttingen, Germany; felix.heinrich@uni-goettingen.de (F.H.); armin.schmitt@uni-goettingen.de (A.O.S.); 2 Center for Integrated Breeding Research (CiBreed), Carl-Sprengel-Weg 1, Georg-August University, 37075 Göttingen, Germany; 3 Faculty of Agriculture, South Westphalia University of Applied Sciences, Lübecker Ring 2, 59494 Soest, Germany

**Keywords:** regulatory SNP, transcription factor, transcription factor binding site, gene regulation, GWAS, database, agricultural plant species, crops

## Abstract

**Simple Summary:**

In breeding research, the investigation of regulatory SNPs (rSNPs) is becoming increasingly important due to their potential causal role for specific functional traits. Especially for crop species, there is still a lack of systematic analyses to detect rSNPs and their predicted effects on the binding of transcription factors. In this study, we present agReg-SNPdb-Plants, a database storing genome-wide collections of regulatory SNPs for agricultural plant species which can be queried via a web interface.

**Abstract:**

Single nucleotide polymorphisms (SNPs) that are located in the promoter regions of genes and affect the binding of transcription factors (TFs) are called regulatory SNPs (rSNPs). Their identification can be highly valuable for the interpretation of genome-wide association studies (GWAS), since rSNPs can reveal the biologically causative variant and decipher the regulatory mechanisms behind a phenotype. In our previous work, we presented agReg-SNPdb, a database of regulatory SNPs for agriculturally important animal species. To complement this previous work, in this study we present the extension agReg-SNPdb-Plants storing rSNPs and their predicted effects on TF-binding for 13 agriculturally important plant species and subspecies (*Brassica napus*, *Helianthus annuus*, *Hordeum vulgare*, *Oryza glaberrima*, *Oryza glumipatula*, *Oryza sativa* Indica, *Oryza sativa* Japonica, *Solanum lycopersicum*, *Sorghum bicolor*, *Triticum aestivum*, *Triticum turgidum*, *Vitis vinifera*, and *Zea mays*). agReg-SNPdb-Plants can be queried via a web interface that allows users to search for SNP IDs, chromosomal regions, or genes. For a comprehensive interpretation of GWAS results or larger SNP-sets, it is possible to download the whole list of SNPs and their impact on transcription factor binding sites (TFBSs) from the website chromosome-wise.

## 1. Introduction

Climate change and its anticipated consequences pose severe challenges to mankind. For agriculture, global warming means that pathogens previously restricted to warmer climates will threaten local animal and plant species as well as expose plants to drought stress due to the increasing water shortage. A rapid and effective adaptation to the new environmental conditions is of paramount importance and can only be achieved through supportive plant breeding programs [1,2]. While breeding once used to be a relatively slow process limited by the generation interval of the species under study, the advent of molecular biology technologies, particularly large-scale genotyping at the whole-genome level, has turned the tide [3,4]. Today, genomic predictions aid the selection process in reproduction, and genome-wide association studies (GWAS) make it possible to identify the genomic loci that are beneficial or deleterious with respect to a trait under study. However, one remaining challenge is to identify not only genomic variants that are statistically associated with a trait, but also those that are actually biologically causative, because this would ensure their efficient use for breeding purposes [5]. In the search for causality of disease- or trait-associated SNPs, one often encounters regulatory SNPs (rSNPs) that influence the amount of genetic material, and hence play a crucial role in the expression of a phenotype. Compared to SNPs in the exonic regions, predicting the consequences of SNPs in the promoter regions is not as straightforward [3,6,7,8]. Such consequences could be the disruption or creation of one or more transcription factor binding sites (TFBSs), which can have a major impact on the level of gene transcription. To date, there exist many tools and databases for the prediction of rSNPs and their impact on regulatory elements such as TFBSs. However, most of them are restricted to the human genome or a few model organisms [9,10,11,12,13,14,15,16,17].

To the best of our knowledge, there exist currently three tools, which generally allow the analysis of plant rSNPs. As a web-based tool, the RSAT variation-tool [18] allows the analysis of user-provided inputs on the fly. However, this tool does not give any information on related genes, as the distance to the transcription start site (TSS) or consequences such as gain- or loss of TFBS, hence the users need to interpret the output themselves. The RSAT variation-tool includes eight crop species and subspecies (*Hordeum vulgare*, *Oryza sativa* Indica, *Oryza sativa* Japonica, *Solanum lycopersicum*, *Sorghum bicolor*, *Triticum turgidum*, *Vitis vinifera*, and *Zea mays*). The R packages MotifbreakR [16] and atSNP [19] principally comprise organisms stored in the Bioconductor BSGenome package [20], which includes only the crop species *Oryza sativa* and *Vitis vinifera*. In both, the user has to provide the SNPs as well as TFBSs (motifs represented as position weight matrices; PWMs) and experience in R programming is imperative.

In our previous studies, we addressed this limited knowledge and created a pipeline for the systematic detection of rSNPs, which we applied to different agriculturally important species such as rapeseed [3], faba bean [7], and various animal species [6]. By creating the database agReg-SNPdb [6], we have provided genome-wide collections of rSNPs for seven different animal species (cattle, pig, chicken, sheep, horse, goat, and dog). In order to extend the available information on rSNPs to additional plant species, we present in this study the database agReg-SNPdb-Plants, which can be considered as an extension of agReg-SNPdb. To the best of our knowledge, agReg-SNPdb-Plants is the first comprehensive database of genome-wide collections of rSNPs and their impact on TFBSs for agriculturally important plant species, which can be queried in various ways: (i) search by SNP ID, (ii) search by chromosomal region, (iii) search by gene, or (iv) a chromosome-wise download of all rSNPs. agReg-SNPdb-Plants includes various important crop species, i.e., Asian rice (Indica and Japonica), barley, bread wheat, durum wheat, grape, maize, rapeseed, sorghum, sunflower, and tomato as well as species, which can serve as genetic resources for the improvement of cultivated species, i.e., African rice and wild rice [21,22]. The availability of rSNPs in rapeseed is particularly noteworthy because to date there exists no genome-wide SNP catalog in Ensembl Plants [23] for this crop. In contrast to the remaining species, where we used the data from Ensembl Plants as basis, we employed a SNP catalog from [24] for rapeseed, which we also used for our previous studies [3,25]. The agReg-SNPdb-Plants web interface is available under https://azifi.tz.agrar.uni-goettingen.de/agreg-snpdb-plants/ (accessed on 24 March 2022).

## 2. Materials and Methods

In our previous work, we have established a pipeline for the detection of rSNPs [6], which requires as input for each species a SNP catalog (as GVF file [26,27]), a reference genome (as fasta file), and gene annotations (as GFF3 file [28]). For all species except for rapeseed, the input data were downloaded from Ensembl Plants [23], with genome assemblies listed in Table 1. The SNP catalog was filtered by removing insertions and deletions as well as SNPs with more than one alternate allele. Since there is no available SNP catalog for rapeseed in Ensembl plants, we used the rapeseed input data from our previous work [3]. This includes a SNP catalog of 670,028 high-quality SNPs (MAF > 0.05) from the cultivars Zhongshuang11 and Zhongyou821 (280 and 133 samples, respectively) collected and published by Lu et al. [24]. The *Brassica napus* reference genome (version 4.1) and gene annotations were obtained from [29] and are available at https://www.genoscope.cns.fr/brassicanapus/data/ (accessed on 3 March 2022).

In brief, the pipeline can be described in the following five steps. For a more detailed description, we refer to [6].

1. **Selection of SNPs in the promoter and surrounding region:** For each gene, we considered a promoter region of 7.5 kb upstream to 2.5 kb downstream from the transcription start site (TSS) and selected all SNPs located within that region. On the website, the user has the possibility to insert a user-defined promoter region with the default being −1 kb to +100 bp.
2. **Extraction of the SNP-flanking region:** Using the reference genomes under study, we extracted 25 bp on each side of a SNP to obtain 51 bp long sequences with the SNP in the central position. During this step, we discarded sequences with a total length of less than 51 bp, sequences containing N’s, and sequences in which the nucleotide at position 26 differed from the reference allele of the SNP (as specified in the SNP catalog in GVF format [26]). The latter only occurred in the species tomato, Asian rice (Indica Group), and sorghum.
3. **Creation of search sequences:** For each SNP, we created an additional copy of its 51 bp long sequence by replacing the reference allele with its alternate allele.
4. **TFBS prediction:** Applying the tool MATCH™ [30] with a plant-specific PWM library containing non-redundant matrices with specific cutoffs that minimize the false positive rate, we predicted TFBSs in the sequences of each SNP. The PWM library is provided by TRANSFAC [31].
5. **Annotation of consequences:** By comparing the two sets of predicted TFBSs, we assessed the consequences of each SNP on a specific TFBS. In particular, the effect of each SNP on a TFBS was assigned to one of the following consequences:
  - Gain of TFBS: the TFBS exists only for the alternate allele of the SNP.
  - Loss of TFBS: the TFBS exists only for the reference allele of the SNP.
  - Score-Change: the TFBS exists for both alleles but with differing binding affinity as determined by the MATCH™ scores.
  - No Change: the TFBS exists for both alleles with the same binding affinity.

## 3. Results

### 3.1. Database

agReg-SNPdb-Plants is centered around four tables: (i) *snp_info* contains general information about the SNPs, (ii) *gene_info* stores general information about the genes, (iii) *snp_region* connects the tables *snp_info* and *gene_info* for all SNPs located in the promoter region of at least one gene, and (iv) *TFBS_results* stores the rSNPs and their consequences with respect to TF-binding. Table 2 shows the numbers of database entries per table and species.

### 3.2. Web Interface

Following the concept of Ensembl and Ensembl Plants, we created an extra web interface for agReg-SNPdb-Plants (https://azifi.tz.agrar.uni-goettingen.de/agreg-snpdb-plants/, accessed on 24 March 2022). The basic functionality was inherited from agReg-SNPdb, e.g., the ability to query the database by searching for (i) SNP identifiers, (ii) SNP position, (iii) chromosomal region, or (iv) gene. Additionally, we enabled the search for several SNP IDs at a time, by pasting white-space separated SNP IDs in the search field.

**Figure 1:**
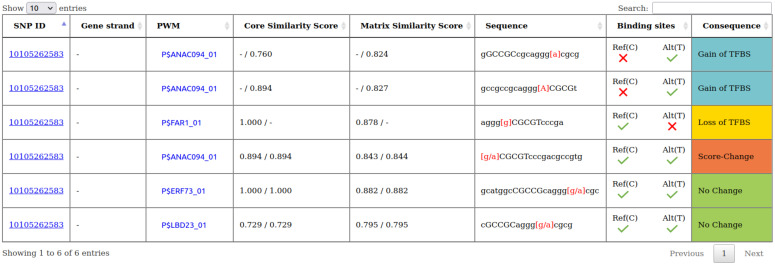
Example of a search result from agReg-SNPdb-Plants showing table *TFBS_results*. The search was performed with the SNP ID 10105262583 from Asian rice (Japonica Group).

Furthermore, we simplified the visualization of the *TFBS_results*, which is shown exemplarily in Figure 1. The first column of table *TFBS_results* (Figure 1) shows the SNP ID. This SNP ID should be the ID as specified in Ensembl Plants. An exception is the naming of the rapeseed SNP IDs, as they are not available in Ensembl Plants we used an annotation as *chr-pos-ref-alt*, e.g., A01-1093-A-G. The second column ‘Gene strand’ refers to the strand of the gene in whose promoter region the SNP is located (the gene strand hence also defines the strand of the sequence). If a SNP occurs in the promoter of two different genes, one on the plus and one on the minus strand, there will be two different tables showing the TFBSs for the plus and minus strands separately. The column ‘PWM’ (position weight matrix) represents the TFBS. The names of the PWMs are defined by TRANSFAC [31] as P$*factorname_version*, where the P$ indicates that the PWM originated from a plant TF and *factorname* specifies the name of the represented TF. The core and matrix similarity scores are the MATCH™ [30] output scores. The ‘Core similarity score’ measures the quality of the match in the first five consecutive most-conserved positions of the PWM and the ‘Matrix similarity score’ measures the quality of the match for the whole PWM. The ‘Sequence’ shows the input sequence matching the PWM with the capital letters representing the core of the PWM and the nucleotides in red representing the SNP position. In case of a loss or gain only the allele for which a TFBS is observed is displayed while in case of a score-change or no change both alleles are displayed. The column ‘Binding site’ is a schematic representation of the column ‘Consequence’, and depicts the presence or absence of a binding site for each allele.

### 3.3. Statistical Overview of the Data

Similar to our previous studies [3,6], we first provide a brief overview of the data stored in agReg-SNPdb-Plants.

**Figure 2:**
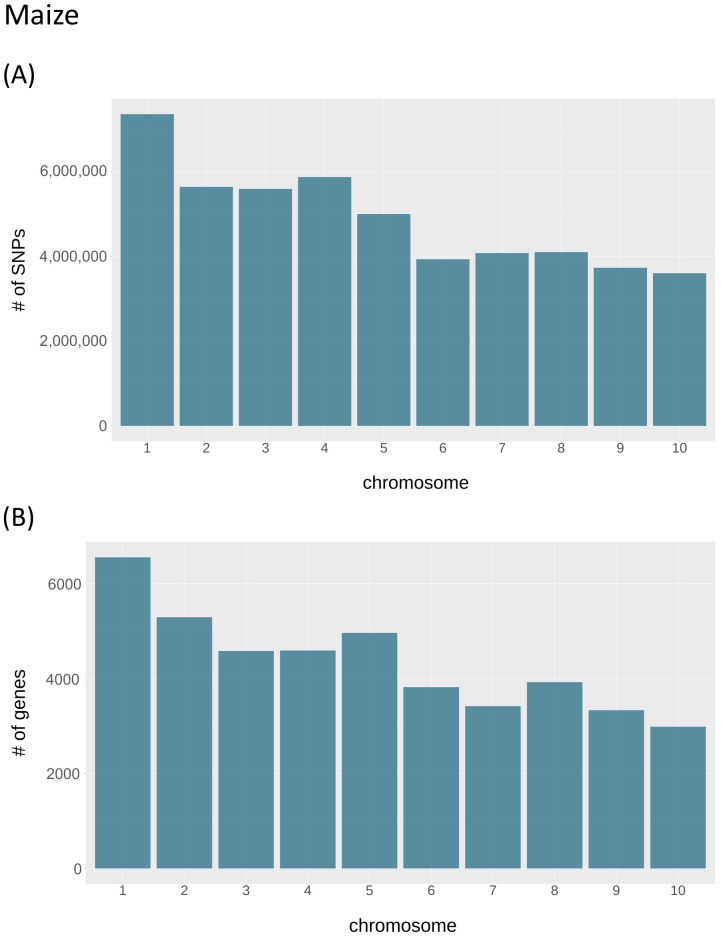
The total number of SNPs and genes per chromosome of maize (*Zea mays*). (**A**) The number of SNPs per chromosome. (**B**) The number of genes per chromosome.

The distributions of SNPs and genes along the chromosomes are exemplary shown for maize (Figure 2; the remaining plots are given in Supplementary Figure S1). As expected, for maize and most other species the absolute numbers of SNPs and genes per chromosomes depend mainly on chromosome size and hence decrease in general with increasing chromosome numbers.

The average number of rSNPs (SNPs that cause a loss or gain of TFBS or a score-change for at least one TFBS) per gene differs strongly across the species. For example, in sunflower we only detected an average of 0.0015 rSNPs per promoter region (−1 kb to +100 bp) while we observed 28.48 rSNPs per promoter in tomato (absolute counts of SNPs and genes for each species can be seen in Table 2). Considering the −1 kb to +100 bp promoter region, on average ~4% of all SNPs are predicted as rSNPs, with a minimum amount of 0.6% in sunflower and a maximum of 13.6% in rapeseed. When examining the number of TFBSs affected by an rSNP, we identified an overall average of ~2 affected TFBSs per rSNP.

**Figure 3:**
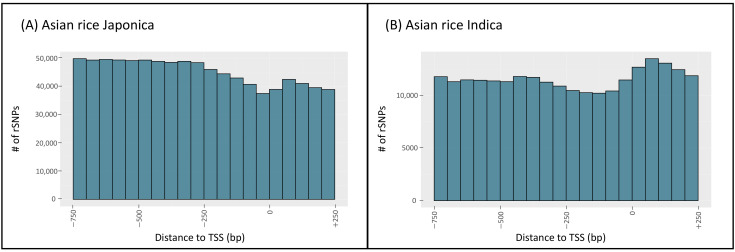
Distribution of the distances between rSNPs and the TSS of (**A**) Asian rice Japonica and (**B**) Asian rice Indica. The histograms show the number of rSNPs in the proximal promoter region (−750 bp to +250 bp) in 50 bp intervals.

To obtain further insights into the data, we investigated the distribution of rSNPs relative to the TSS (Supplementary Figures S2). Similar to the animal species in agReg-SNPdb, we observed two different patterns for the distributions. The first pattern shows that the sequence is protected from variations in close proximity to the TSS, while the number of rSNPs increases with increasing distance in the upstream direction [3,6,32]. A similar pattern was observed in rapeseed, barley, Asian rice Japonica, maize, tomato, wild rice, and sorghum (Figure 3A and Figure S2). The second pattern shows the opposite: The number of rSNPs increases with increasing downstream distance. This was observed in sunflower, African rice, Asian rice Indica, bread wheat, durum wheat, and grape (Figure 3B and Figure S2). Figure 3 exemplary shows the comparison of the rSNP distance to the TSS for the two types of *Oryza sativa*, Japonica in (A) and Indica in (B).

## 4. Discussion

Transcription factors bind to the promoter region to fine-tune the level of gene expression in all higher organisms. A regulatory SNP within a TFBS can influence this transcriptional gene regulation to a great extent and hence could have a causative effect on the phenotype. In plants, several studies investigated (single) rSNPs with respect to a specific trait or phenotype [3,7,33,34,35]. For example, Konishi et al. revealed an rSNP in rice that causes a loss of TFBS for an ABI3 type TF in the promoter region of the quantitative trait locus (QTL) for seed shattering on chromosome 1 (*qSH1*). This rSNP is causative for the loss of seed shattering and thus paved the way for rice domestication [35]. In maize, several rSNPs were detected in the promoter of the maize rough dwarf disease candidate gene eukaryotic translation initiation factor 4E (*eIF4E*) and control its expression level [34]. Furthermore, in wheat, an rSNP associated with wheat grain weight affects the binding of a calmodulin-binding TF and hence the gene expression of the *TaGW2-6A* gene, a candidate gene for grain weight [33]. Similar to these studies, in our previous study on the grain legume faba bean we discovered two rSNPs which are significantly associated with the vicine and convicine content and affect the binding of the TFs MYB4, MYB61, and SQUA [7]. To this end, we have investigated the seed oil content in rapeseed of the cultivars Zhongshuang11 and Zhongyou821 and obtained a genome-wide collection of rSNPs which are significantly associated with the oil content and positioned in promoter regions of genes differentially expressed between high and low oil content cultivars [3].

Due to the increasing interest in finding causative rSNPs yet limited availability of resources to detect rSNPs in crop species, we used our rSNP detection pipeline to systematically analyze 13 crop plants and provide a database of genome-wide rSNPs which can be queried via a web interface (https://azifi.tz.agrar.uni-goettingen.de/agreg-snpdb-plants/, accessed on 24 March 2022). This pipeline could be highly valuable for scientists to interpret their results from e.g., a GWAS or next generation sequencing (NGS) experiments.

In our pipeline, one important step was the selection of the range of the promoter regions, since this determines if a SNP is considered for further analyses. Even though the core promoter is considered to be positioned within ~200 bp around the TSS [32], a wider promoter region can be targeted by TFs to regulate gene transcription. Previous studies defined different promoter regions for TFBS prediction, ranging from −10 kb to +10 kb [6,13,14,36,37,38,39,40,41,42] (the different promoter definitions and respective textual evidences are provided in Table S1). Therefore, we used a relatively wide promoter region ranging from −7.5 kb to +2.5 kb relative to the TSS, in order to ensure the inclusion of the regulatory regions. However, it is important to note that the biological promoter is usually smaller and, hence, our web interface provides the possibility to select a smaller user-defined promoter region.

In total, we analyzed 13 species and subspecies for the construction of the agReg-SNPdb-Plants database, for twelve of which reference genome, gene annotations, and a SNP catalog were available in Ensembl Plants.

However, for some species the available information, e.g., the reference genome, might not be of the same quality compared to other, well-investigated species. Furthermore, due to the amount of repetitive sequences in some plant species such as bread wheat or maize, both the reference genome annotation as well as locating genomic variants can be challenging [43,44]. The quality of the promoter region highly influences the quality of TFBS predictions and we want to emphasize that our predictions can only rely on the available information. For the species tomato, Asian rice (Indica), and sorghum, we observed that the alleles of several SNPs do not fit to the reference genome, in particular, their reference alleles were not present at the SNP position in the reference genome. An example for this issue, can be shown based on the tomato SNP vcZYOCUX (T/A), where the base at the respective position in the reference genome is G (https://plants.ensembl.org/Solanum_lycopersicum/Variation/Explore?r=1:39003479-39004479;v=vcZYOCUX;vdb=variation;vf=3506065, accessed on 24 March 2022). Such issues indicate that there is still a need for further investigation or updates to improve the genome sequences as well as SNP annotations. In our pipeline, we excluded such SNPs from further analysis to ensure the highest possible reliability of our results.

## 5. Conclusions

In breeding research, the knowledge about rSNPs can help to unravel the regulatory mechanisms underlying specific phenotypes and could hence lead to the identification of causal SNPs, which are of great importance for the establishment of robust markers. To the best of our knowledge, until now there exists no database storing genome-wide rSNPs and their consequences on TF binding in plant sciences which can be queried in various ways. In order to address this lack of information, and thus complementing our previous work, we created agReg-SNPdb-Plants, a database of rSNPs for 13 agricultural plant species and subspecies with currently available SNP annotations. Its web interface is a helpful resource for scientists who are conducting association analyses such as GWAS, gene expression experiments, expression QTL (eQTL) studies, or population studies. Consequently, they can automatically investigate the candidate SNPs or specific genes to rate them by their importance or causality. In this regard, our user interface provides different search functions and delivers information on the consequences of rSNPs on TF binding such as (i) gain of TFBS, (ii) loss of TFBS, (iii) change of binding affinity, or (iv) no change. Due to regular updates of genomes, gene- and SNP-annotations, our database will be regularly updated to add new plant species when available and to update existing ones.

## Figures and Tables

**Table 1:** Assembly versions of the input data from Ensembl Plants including reference genome, SNP catalog and gene annotations.

Plant	Assembly Version	Download Date (DD/MM/YYYY)
*Helianthus annuus* (sunflower)	HanXRQr1.0	11/08/2021
*Hordeum vulgare* (barley)	MorexV3_pseudomolecules_assembly	12/22/2021
*Oryza glaberrima* (African rice)	Oryza_glaberrima_V1	11/08/2021
*Oryza glumipatula* (wild rice)	Oryza_glumaepatula_v1.5	11/08/2021
*Oryza sativa* Indica (Asian rice Indica)	ASM465v1	12/22/2021
*Oryza sativa* Japonica (Asian rice Japonica)	IRGSP-1.0	11/08/2021
*Solanum lycopersicum* (tomato)	SL3.0	12/22/2021
*Sorghum bicolor* (sorghum)	Sorghum_bicolor_NCBIv3	12/22/2021
*Triticum aestivum* (bread wheat)	IWGSC	11/08/2021
*Triticum turgidum* (durum wheat)	Svevo.v1	11/08/2021
*Vitis vinifera* (grape)	12X	11/08/2021
*Zea mays* (maize)	Zm-B73-REFERENCE-NAM-5.0	11/08/2021

**Table 2:** The number of records stored in the database tables *snp_info*, *gene_info*, *snp_region*, and *TFBS_results* separated by species.

Plant	*snp_info*	*gene_info*	*snp_region*	*TFBS_results*
African rice	7,567,669	33,164	7,341,550	8,336,778
Asian rice Indica	4,340,785	37,878	4,589,915	4,441,820
Asian rice Japonica	25,135,669	37,960	20,155,983	20,940,720
Barley	12,771,762	35,106	2,545,069	2,736,205
Bread wheat	18,093,867	107,889	13,334,911	19,733,723
Durum wheat	1,815,904	66,559	1,121,107	1,734,495
Grape	400,940	29,971	334,500	290,793
Maize	48,830,598	44,289	15,439,220	13,101,269
Rapeseed	670,028	406,325	5,110,349	506,859
Sorghum	8,081,051	34,023	6,414,543	3,118,613
Sunflower	11,834	52,191	2335	1498
Tomato	60,973,560	33,869	28,709,218	10,347,415
Wild rice	4,865,161	35,735	4,752,796	5,154,313
Total	193,558,828	954,959	109,851,496	90,444,501

## Data Availability

https://azifi.tz.agrar.uni-goettingen.de/agreg-snpdb-plants/ (accessed on 24 March 2022).

## Supplementary Materials

The following supporting information can be downloaded at: https://www.mdpi.com/article/10.3390/biology11050684/s1, Figure S1: Histograms of the total numbers of SNPs and genes per chromosome for each plant stored in agReg-SNPdb-Plants, Figure S2: Distribution of rSNPs around the TSS for each plant stored in agReg-SNPdb-Plants, Table S1: Textual evidences of different promoter definitions used by different studies for TFBS prediction or similar analyses.

## Abbreviations

The following abbreviations are used in this manuscript:

SNP	single nucleotide polymorphism
rSNP	regulatory SNP
TF	transcription factor
TFBS	transcription factor binding site
TSS	transcription start site
bp	base pair
GWAS	genome-wide association study
eQTL	expression quantitative trait locus
PWM	position weight matrix
NGS	next generation sequencing
MAF	minor allele frequency
