# Supplementary material for: agReg-SNPdb-Plants: A Database of Regulatory SNPs for Agricultural Plant Species
Source: Biology (Basel). 2022 Apr 29;11(5):684. doi: 10.3390/biology11050684 (PMC9138521; doi:10.3390/biology11050684)
Supplement: Supplementary file 1 [file biology-11-00684-s001.zip › Suppl_FiguresS1_SNPs_and_genes.pdf]

Supplementary Figures S1: Histograms of the total numbers of SNPs and genes per chromosome for each plant stored in agReg-SNPdb-Plants.

African rice

(A)

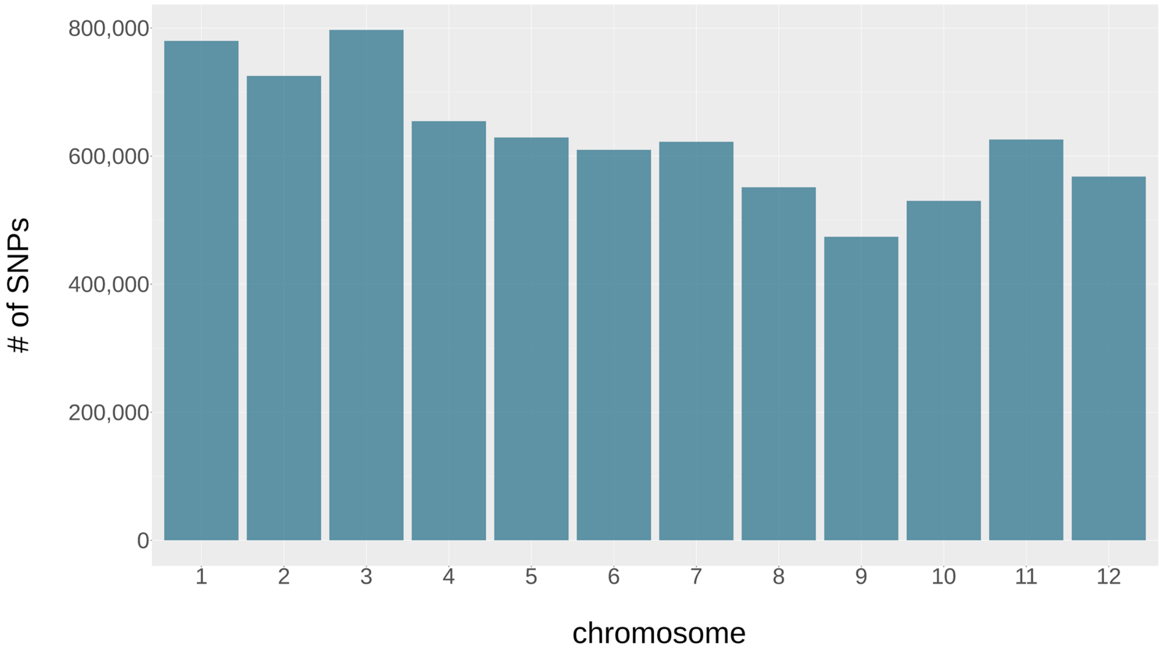

(B)

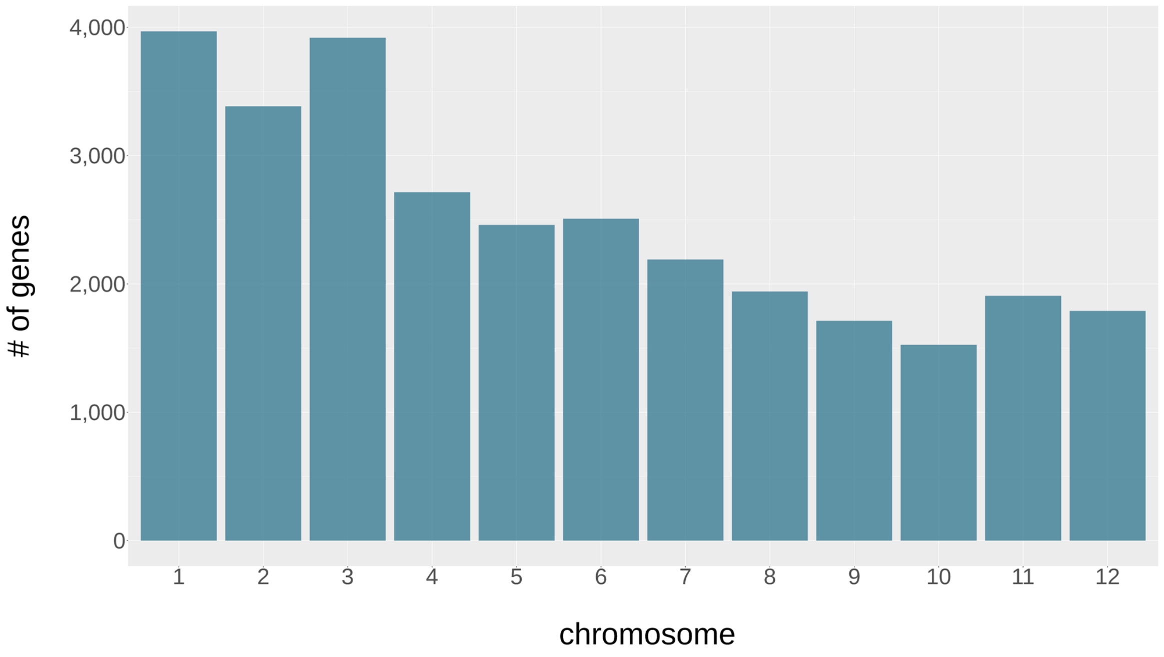

Figure S1.1: Total number of SNPs and genes for each chromosome of *Oryza glaberrima* (African rice). (A) shows the number of SNPs per chromosome. (B) shows the number of genes per chromosome. For this plot, only the main chromosomes are included, excluding all unmapped contigs/scaffolds, random and mitochondrial chromosomes.

Asian rice Indica

(A)

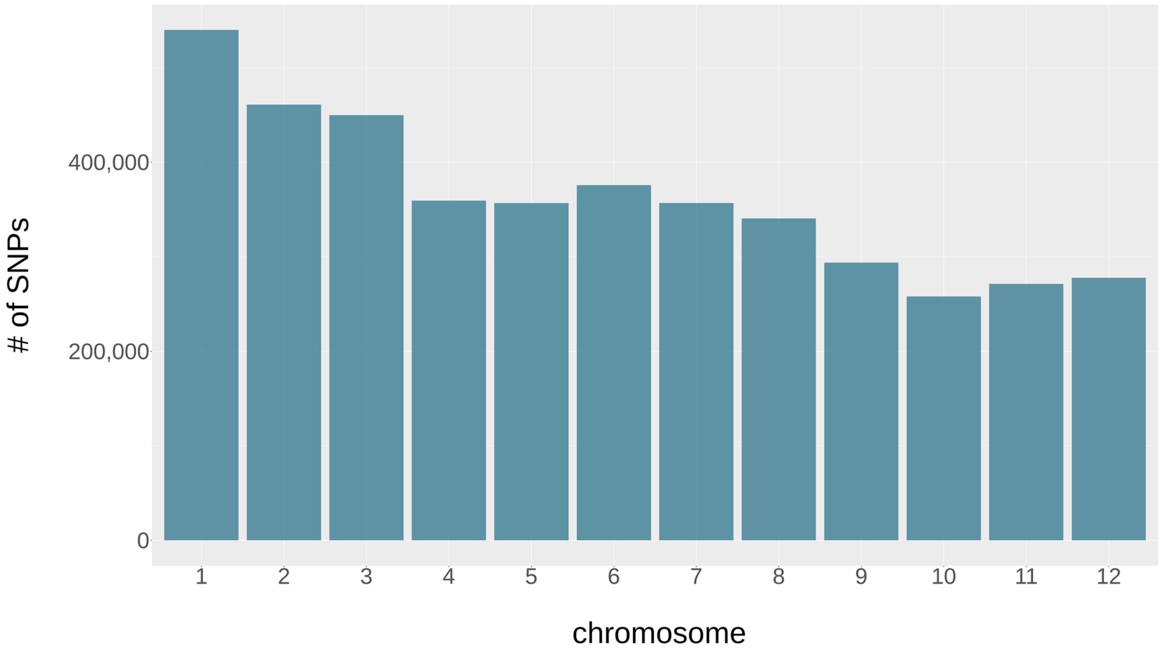

(B)

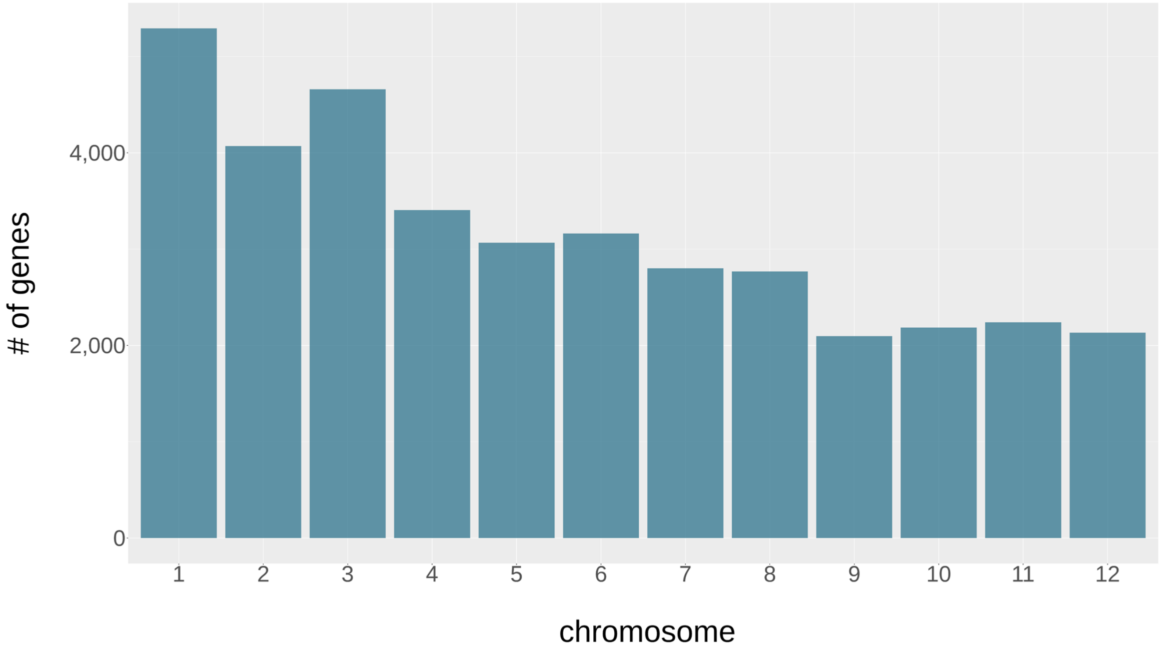

Figure S1.2: Total number of SNPs and genes for each chromosome of Asian rice Indica (*Oryza sativa* Indica). (A) shows the number of SNPs per chromosome. (B) shows the number of genes per chromosome. For this plot, only the main chromosomes are included, excluding all unmapped contigs/scaffolds, random and mitochondrial chromosomes.

Asian rice Japonica

(A)

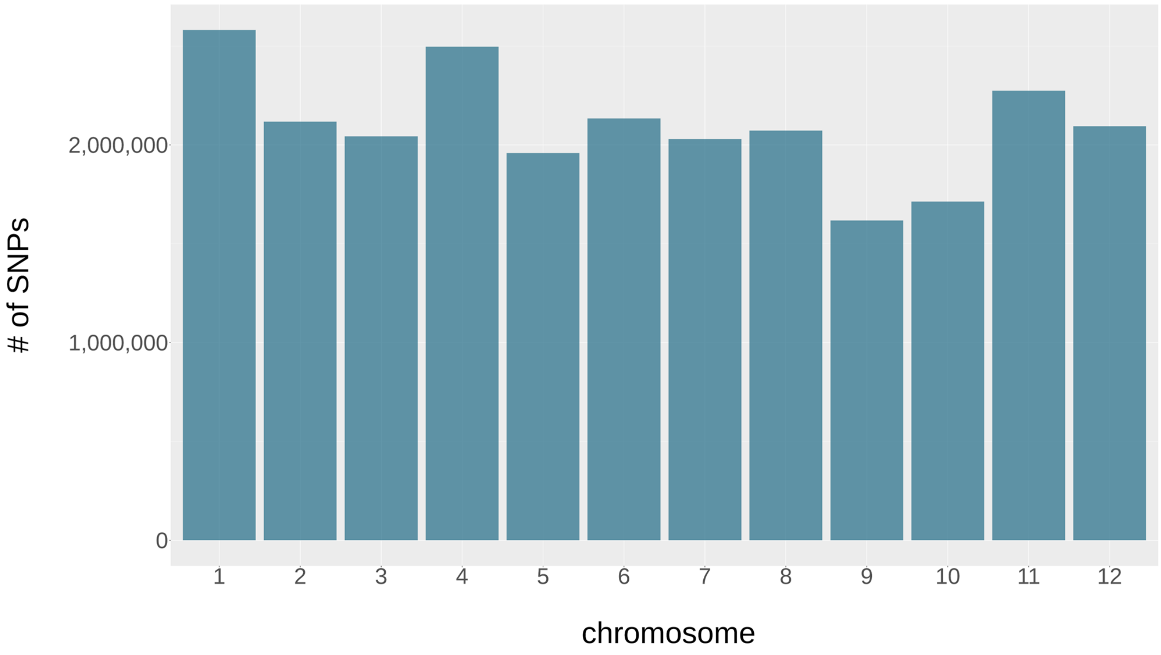

(B)

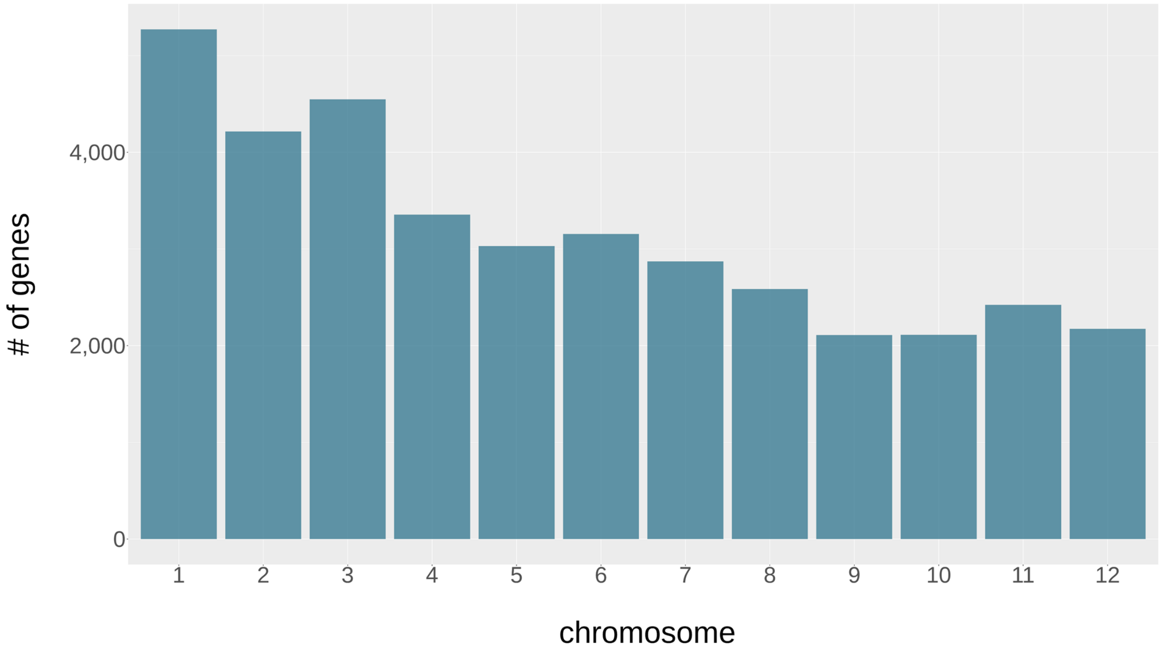

Figure S1.3: Total number of SNPs and genes for each chromosome of Asian rice Japonica (*Oryza sativa* Japonica). (A) shows the number of SNPs per chromosome. (B) shows the number of genes per chromosome. For this plot, only the main chromosomes are included, excluding all unmapped contigs/scaffolds, random and mitochondrial chromosomes.

Barley

(A)

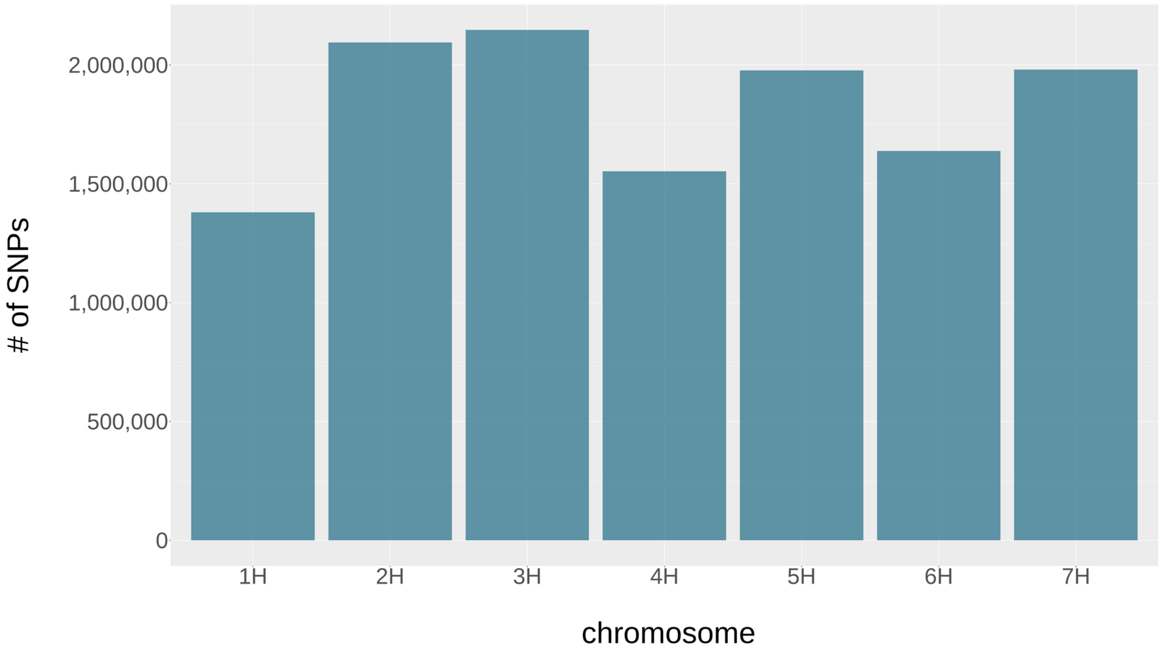

(B)

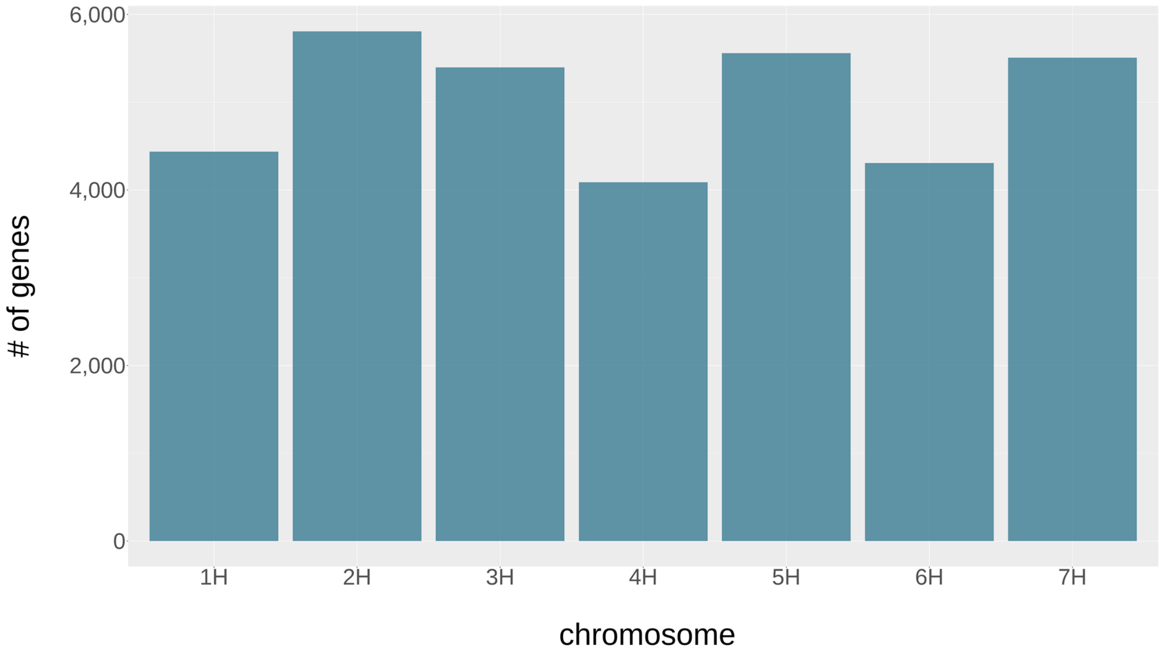

Figure S1.4: Total number of SNPs and genes for each chromosome of barley (*Hordeum vulgare*). (A) shows the number of SNPs per chromosome. (B) shows the number of genes per chromosome. For this plot, only the main chromosomes are included, excluding all unmapped contigs/scaffolds, random and mitochondrial chromosomes.

Bread wheat

(A)

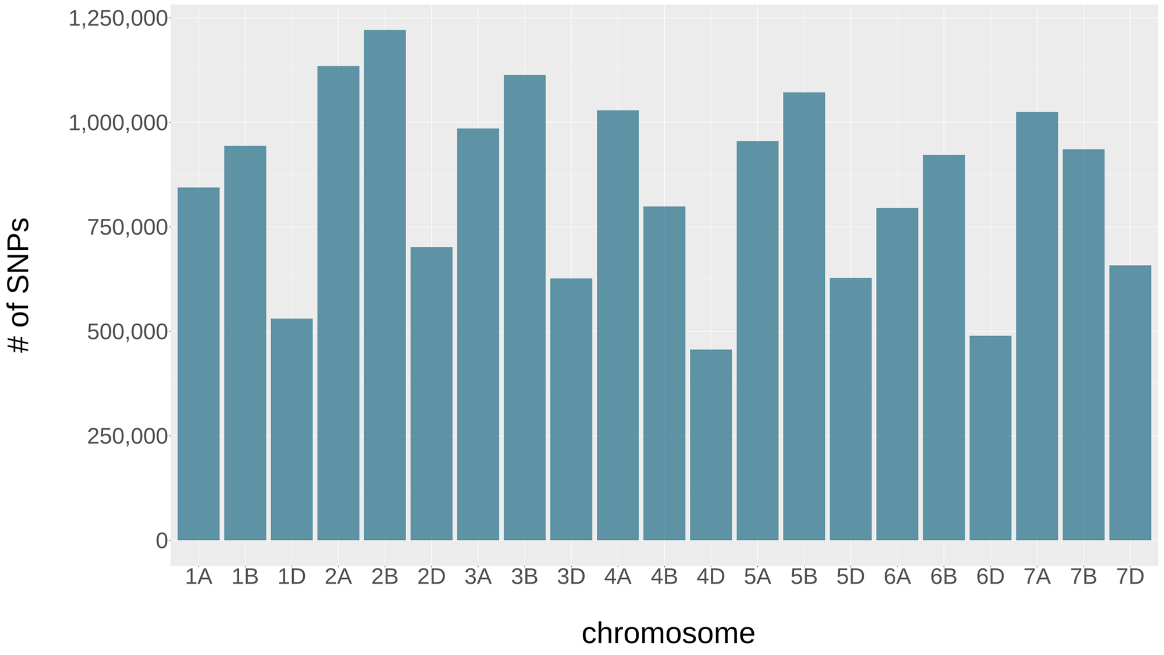

(B)

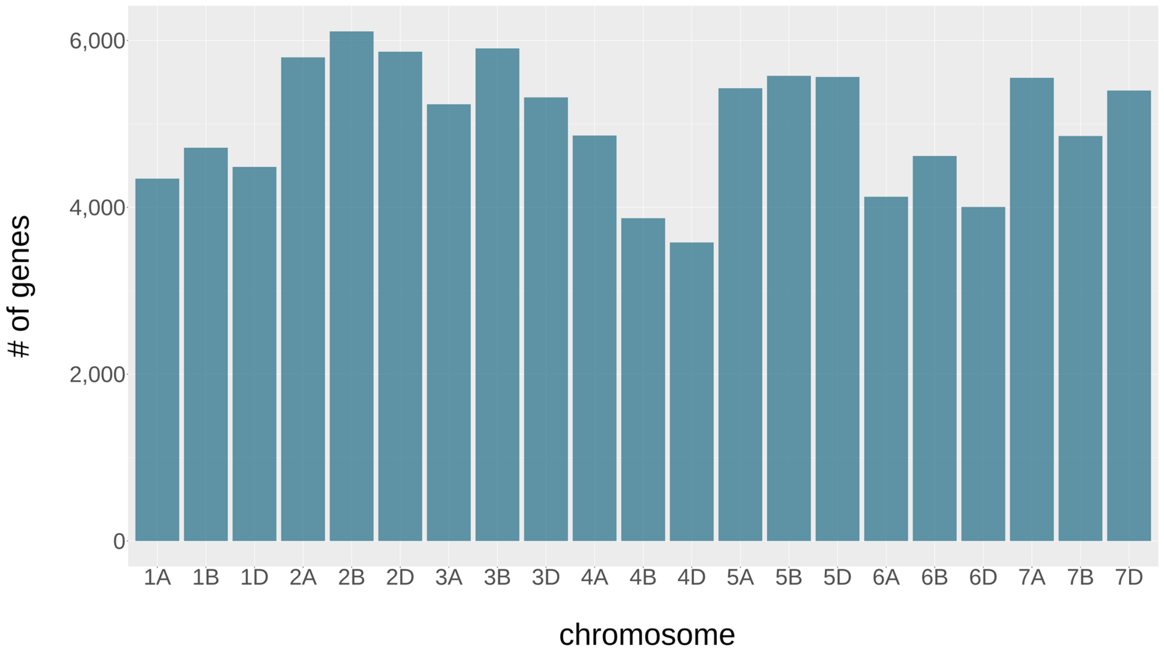

Figure S1.5: Total number of SNPs and genes for each chromosome of bread wheat (*Triticum aestivum*). (A) shows the number of SNPs per chromosome. (B) shows the number of genes per chromosome. For this plot, only the main chromosomes are included, excluding all unmapped contigs/scaffolds, random and mitochondrial chromosomes.

Durum wheat

(A)

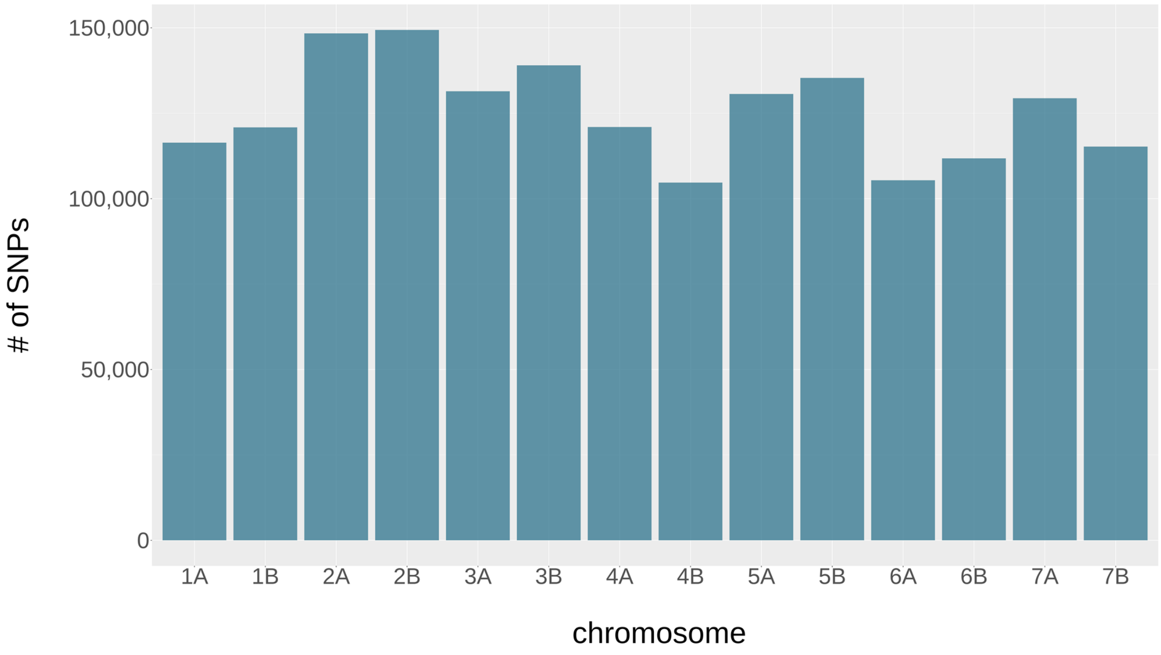

(B)

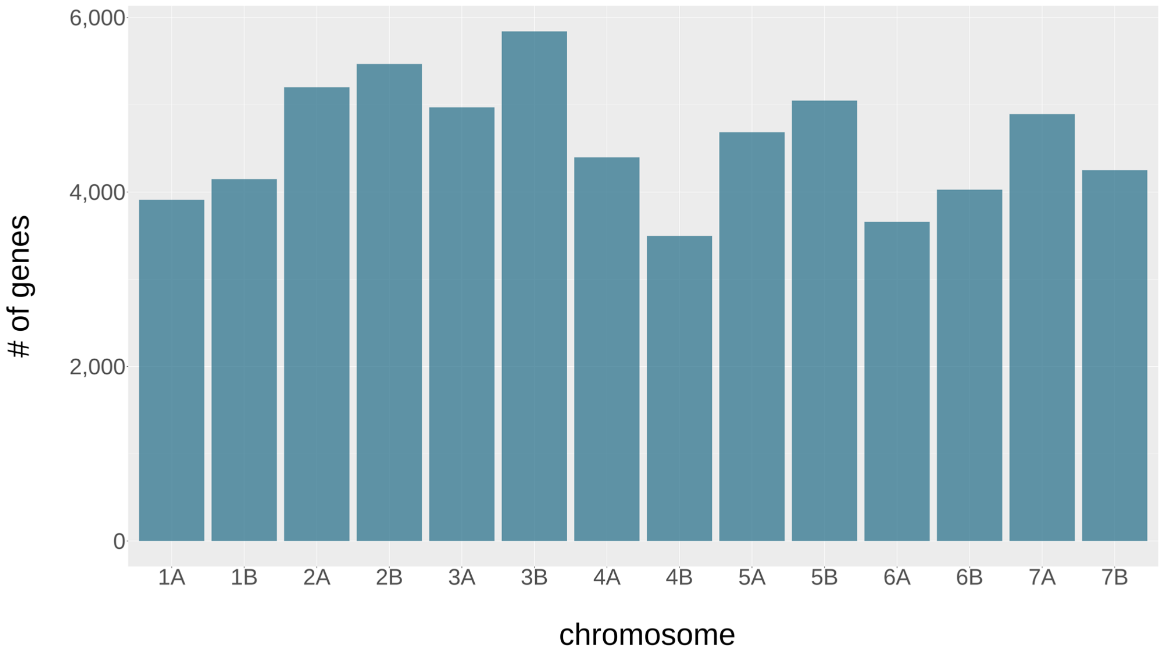

Figure S1.6: Total number of SNPs and genes for each chromosome of durum wheat (*Triticum turgidum*). (A) shows the number of SNPs per chromosome. (B) shows the number of genes per chromosome. For this plot, only the main chromosomes are included, excluding all unmapped contigs/scaffolds, random and mitochondrial chromosomes.

Grape

(A)

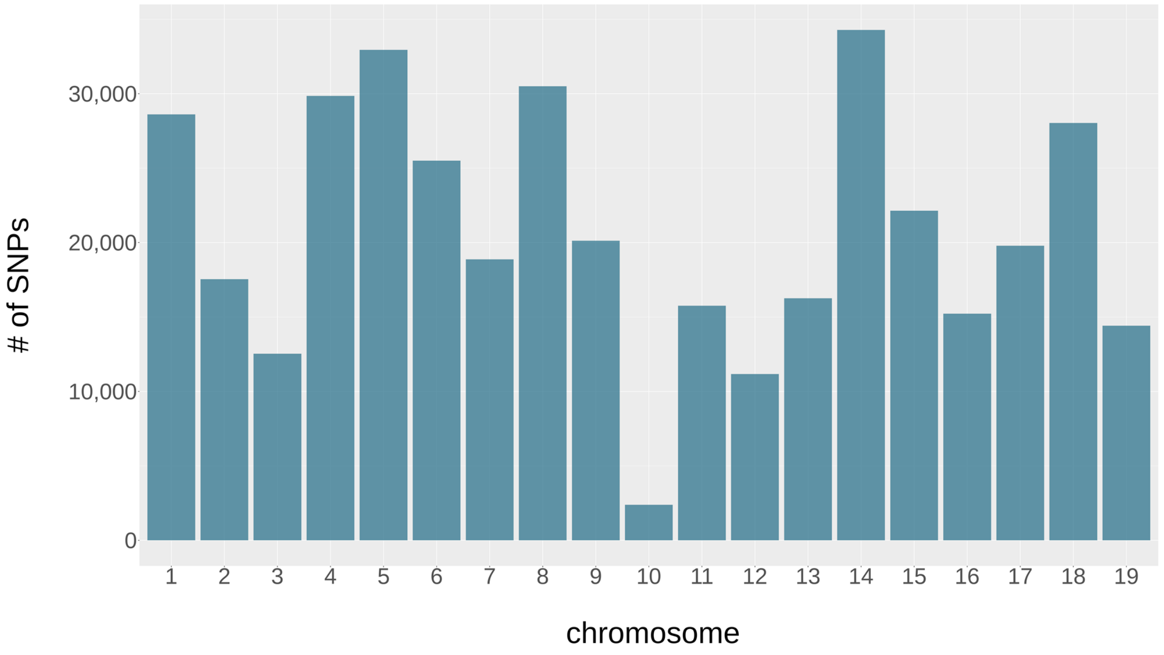

(B)

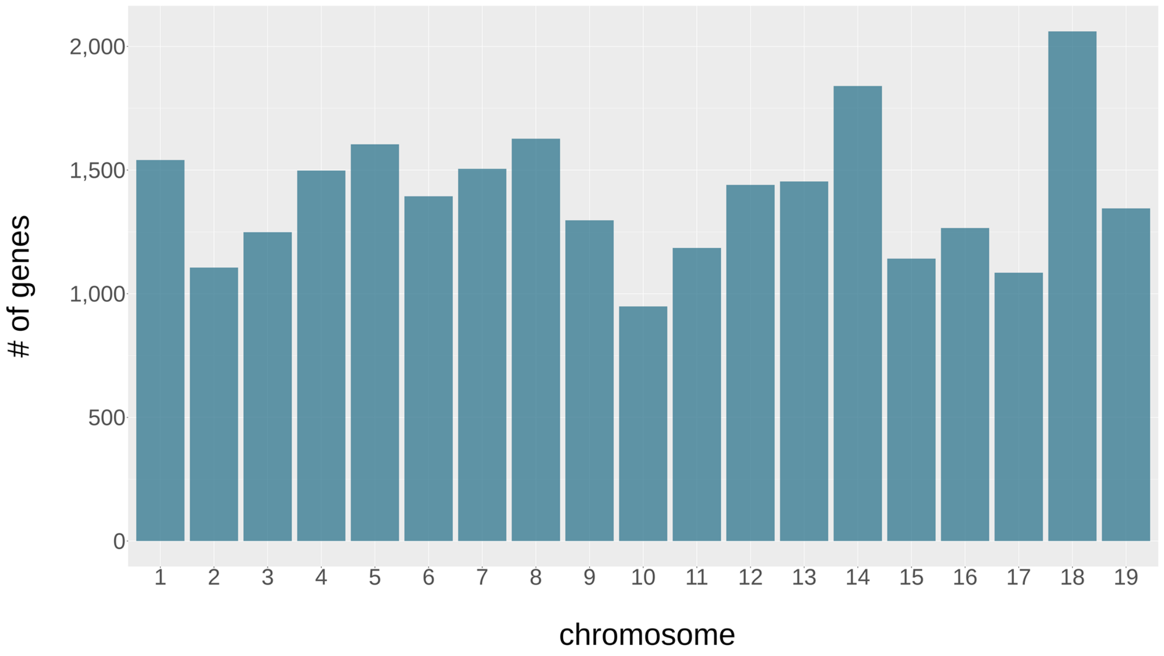

Figure S1.7: Total number of SNPs and genes for each chromosome of grape (*Vitis vinifera*). (A) shows the number of SNPs per chromosome. (B) shows the number of genes per chromosome. For this plot, only the main chromosomes are included, excluding all unmapped contigs/scaffolds, random and mitochondrial chromosomes.

Maize

(A)

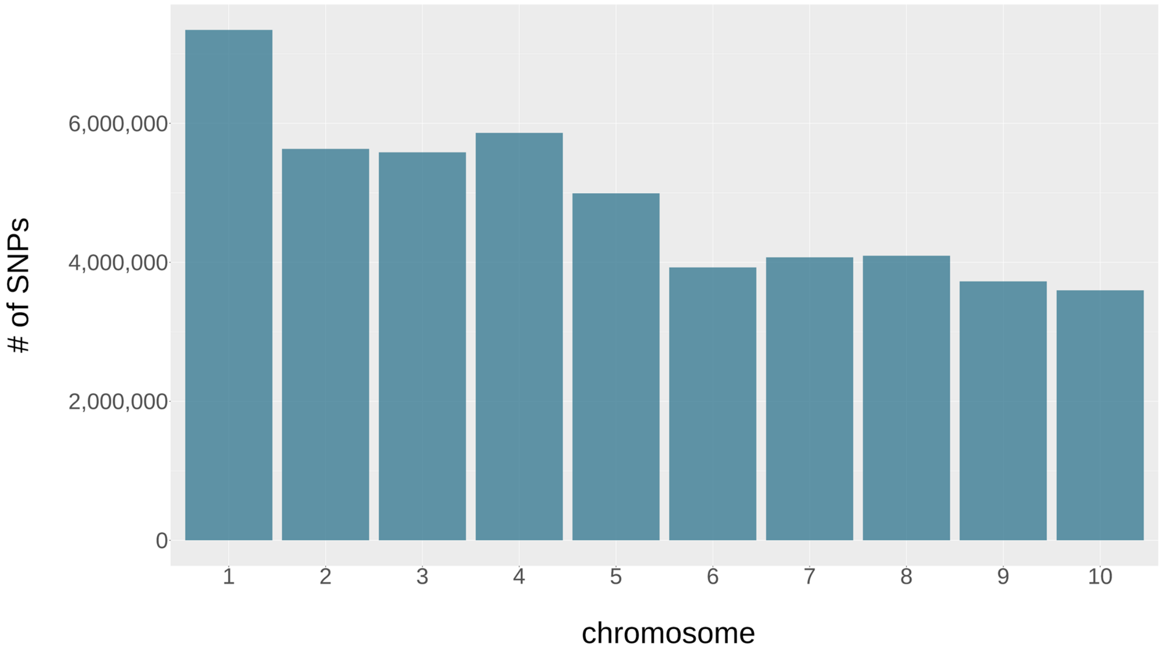

(B)

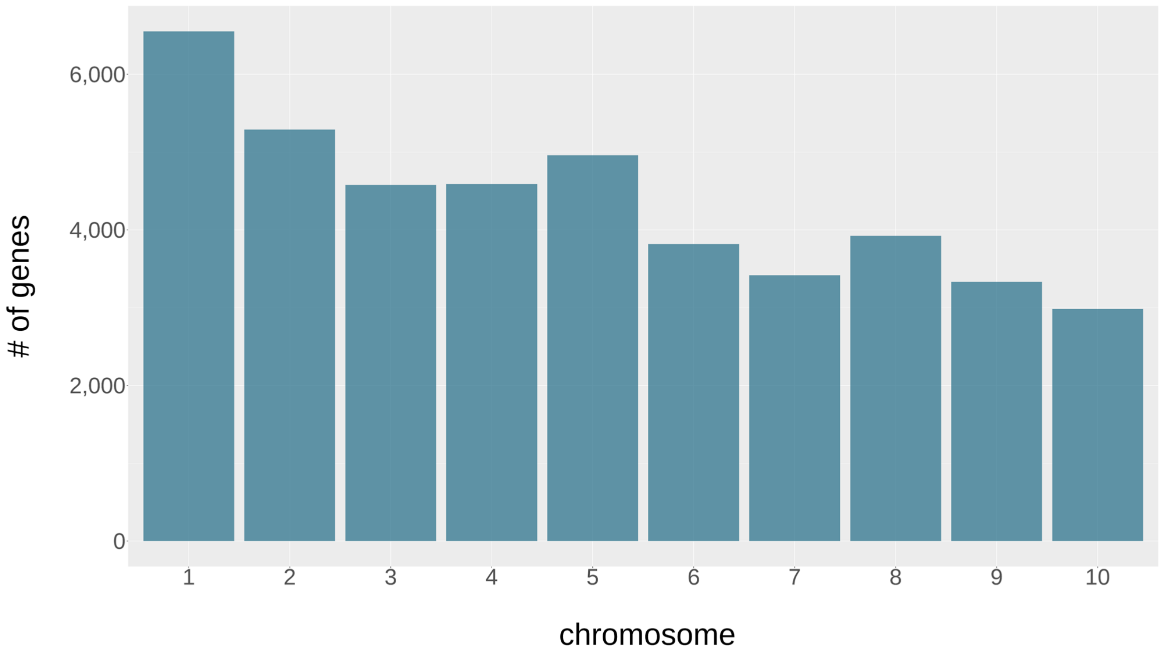

Figure S1.8: Total number of SNPs and genes for each chromosome of maize (*Zea mays*). (A) shows the number of SNPs per chromosome. (B) shows the number of genes per chromosome. For this plot, only the main chromosomes are included, excluding all unmapped contigs/scaffolds, random and mitochondrial chromosomes.

Rapeseed

(A)

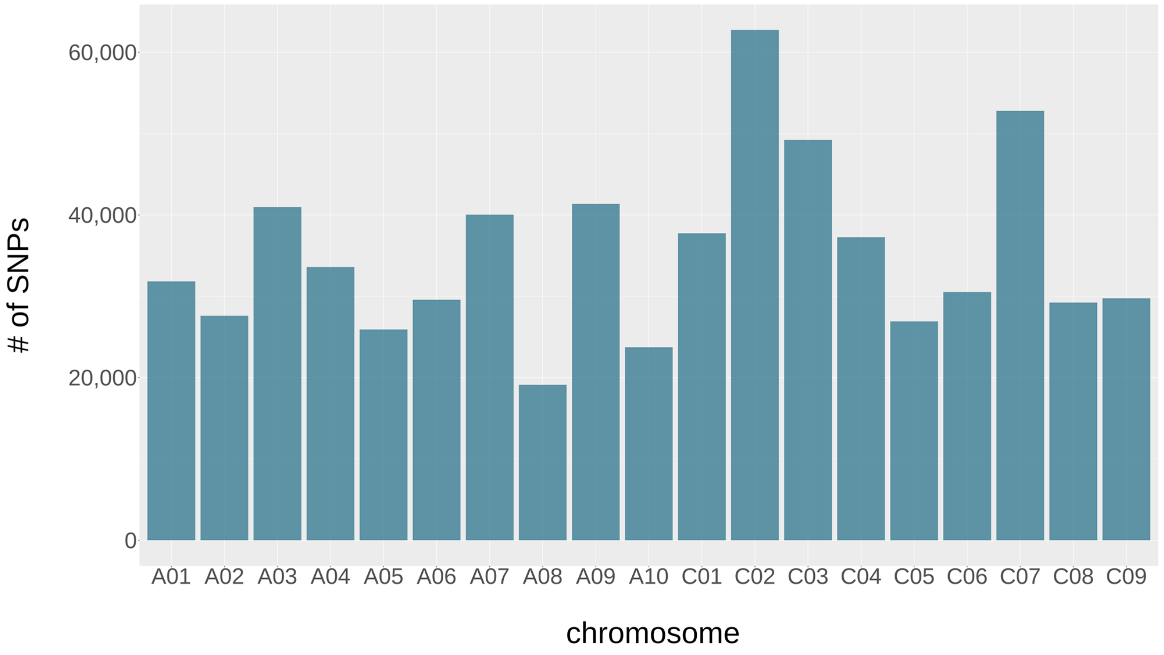

(B)

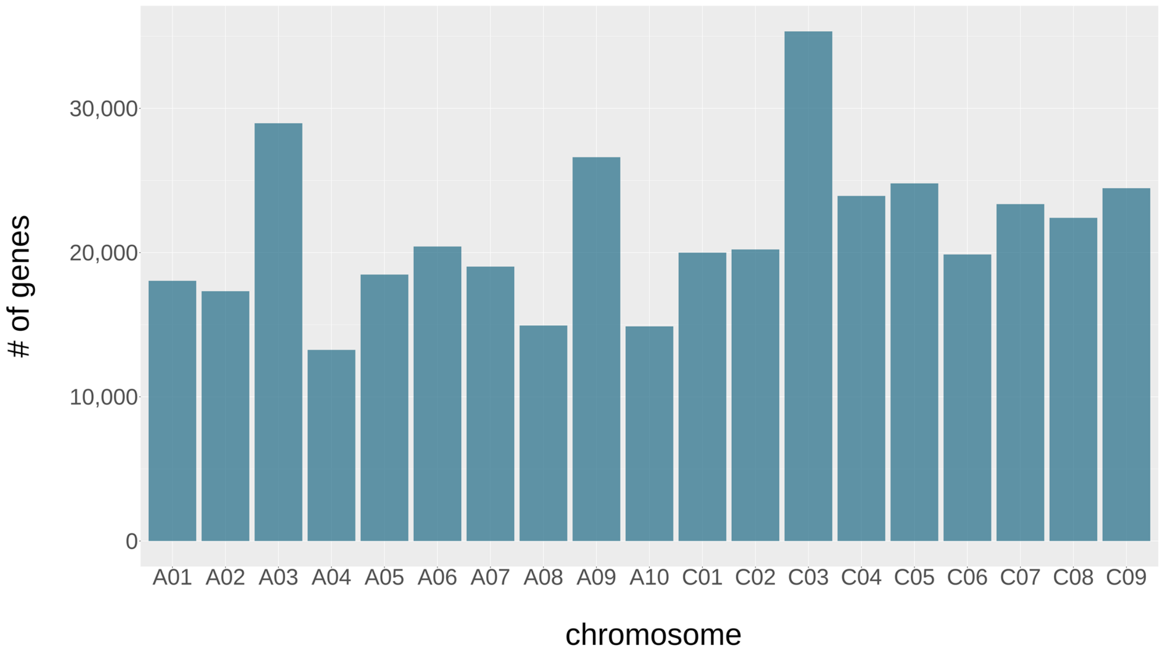

Figure S1.9: Total number of SNPs and genes for each chromosome of rapeseed (*Brassica napus*). (A) shows the number of SNPs per chromosome. (B) shows the number of genes per chromosome. For this plot, only the main chromosomes are included, excluding all unmapped contigs/scaffolds, random and mitochondrial chromosomes.

Sorghum

(A)

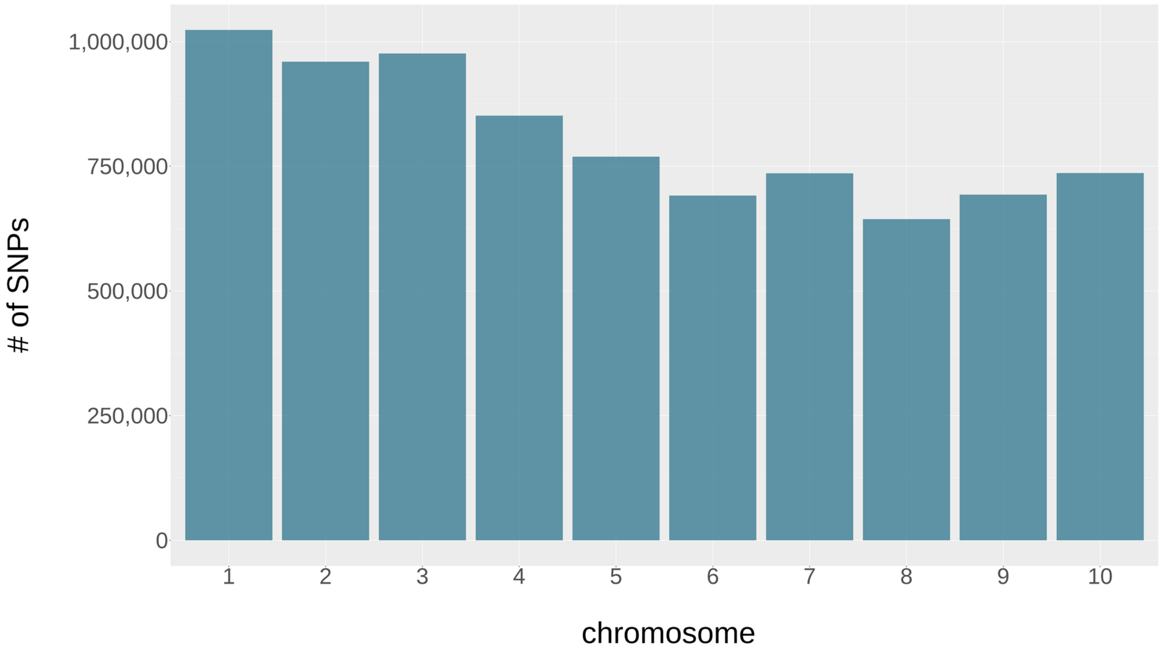

(B)

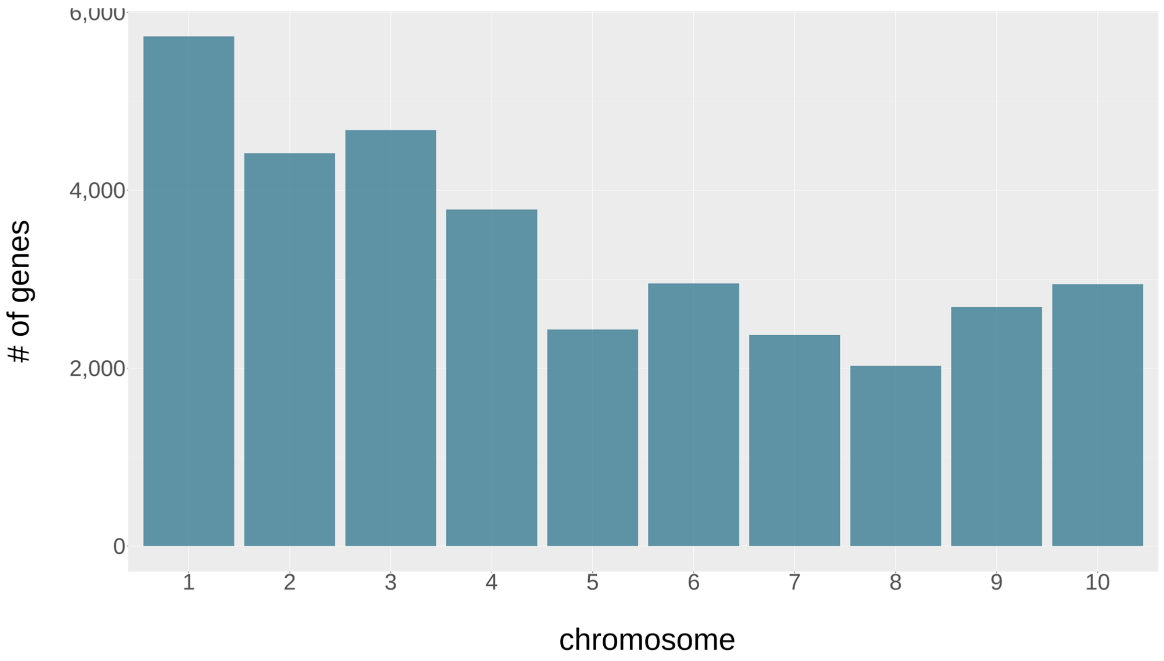

Figure S1.10: Total number of SNPs and genes for each chromosome of sorghum (*Sorghum bicolor*). (A) shows the number of SNPs per chromosome. (B) shows the number of genes per chromosome. For this plot, only the main chromosomes are included, excluding all unmapped contigs/scaffolds, random and mitochondrial chromosomes.

Sunflower

(A)

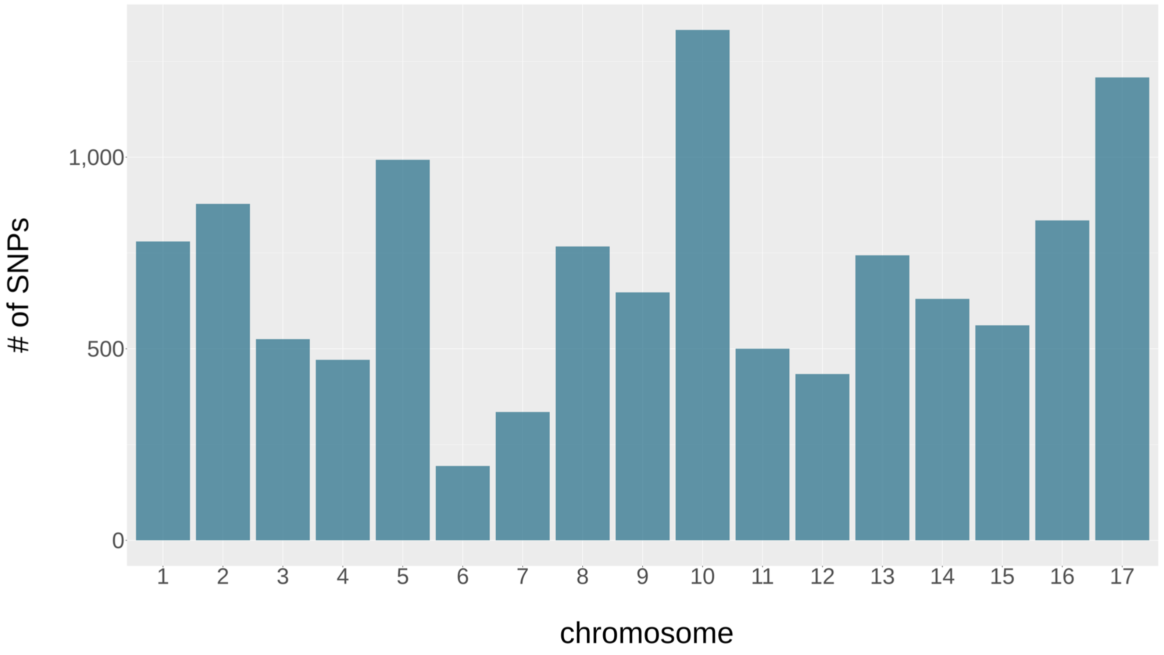

(B)

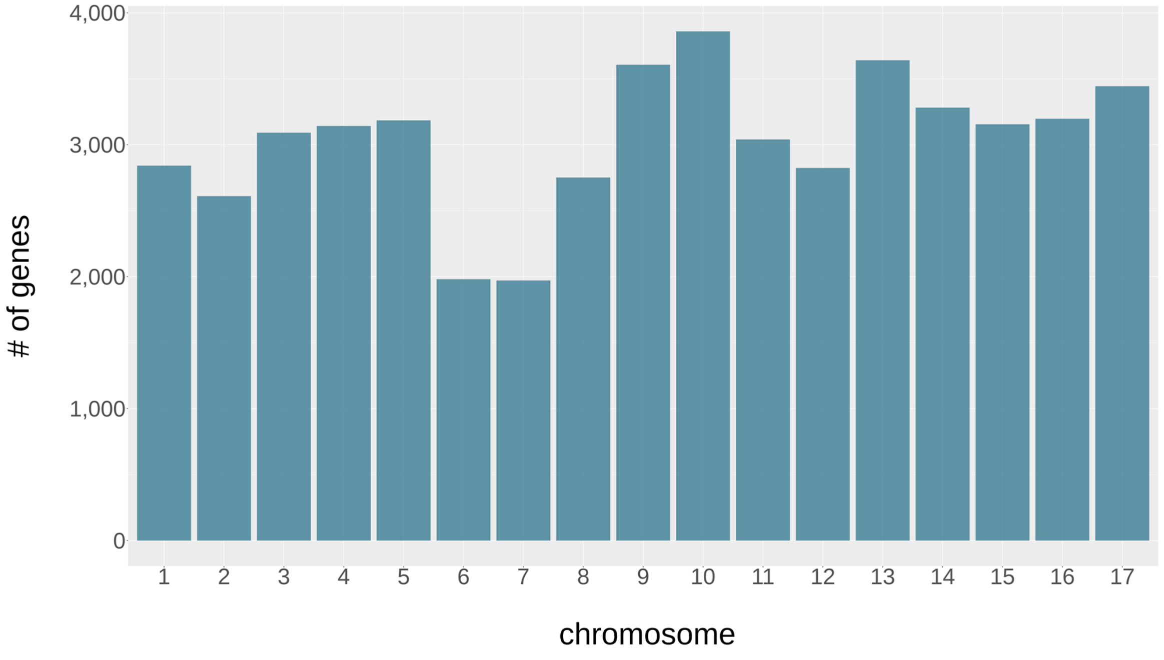

Figure S1.11: Total number of SNPs and genes for each chromosome of sunflower (*Helianthus annuus*). (A) shows the number of SNPs per chromosome. (B) shows the number of genes per chromosome. For this plot, only the main chromosomes are included, excluding all unmapped contigs/scaffolds, random and mitochondrial chromosomes.

Tomato

(A)

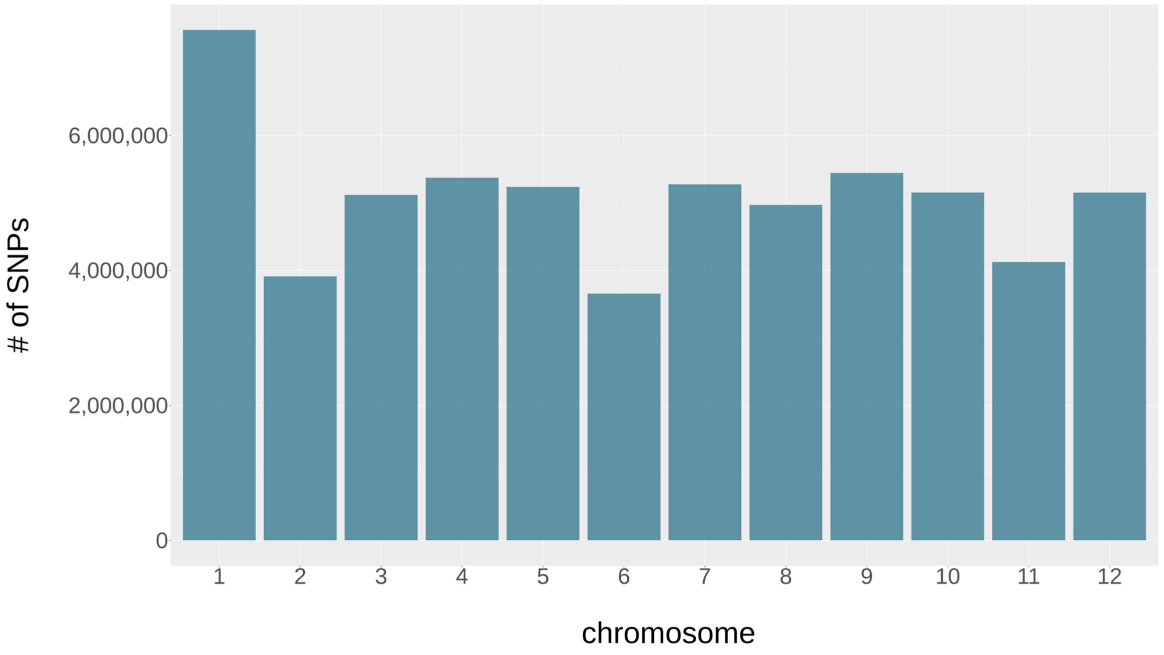

(B)

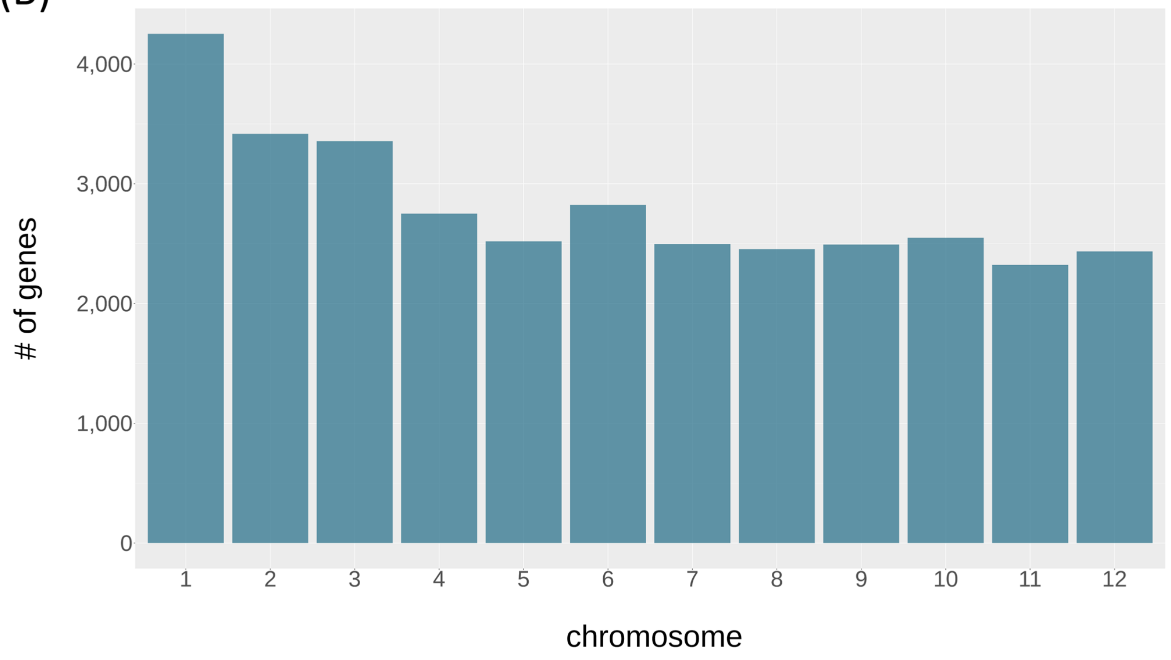

Figure S1.12: Total number of SNPs and genes for each chromosome of tomato (*Solanum lycopersicum*). (A) shows the number of SNPs per chromosome. (B) shows the number of genes per chromosome. For this plot, only the main chromosomes are included, excluding all unmapped contigs/scaffolds, random and mitochondrial chromosomes.

Wild rice

(A)

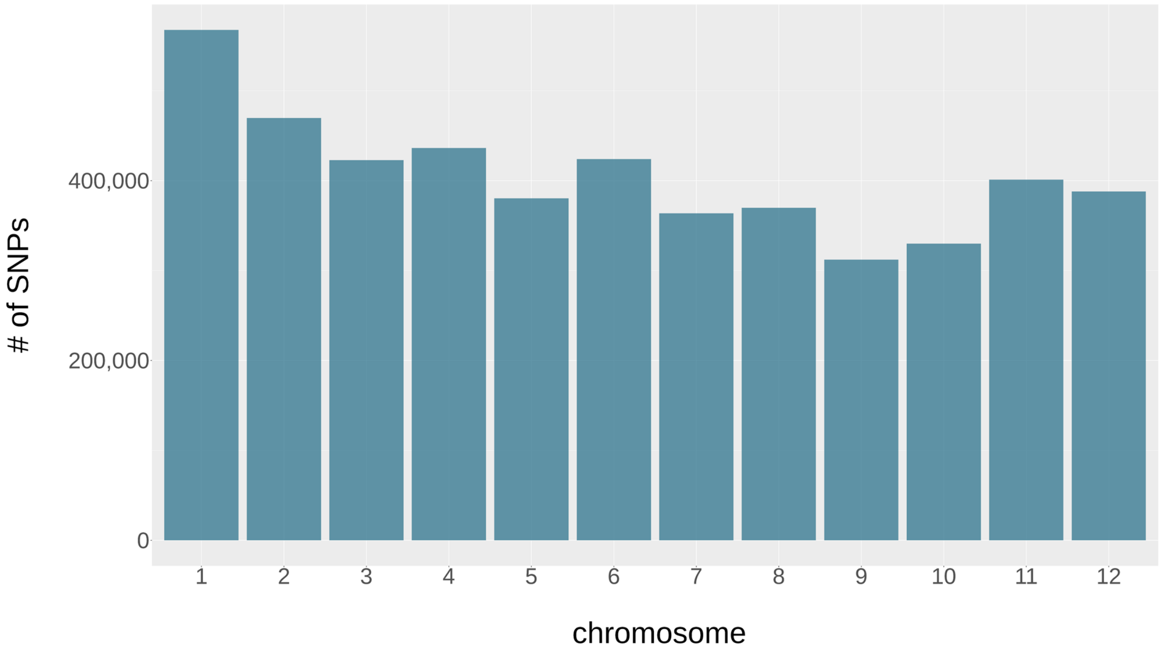

(B)

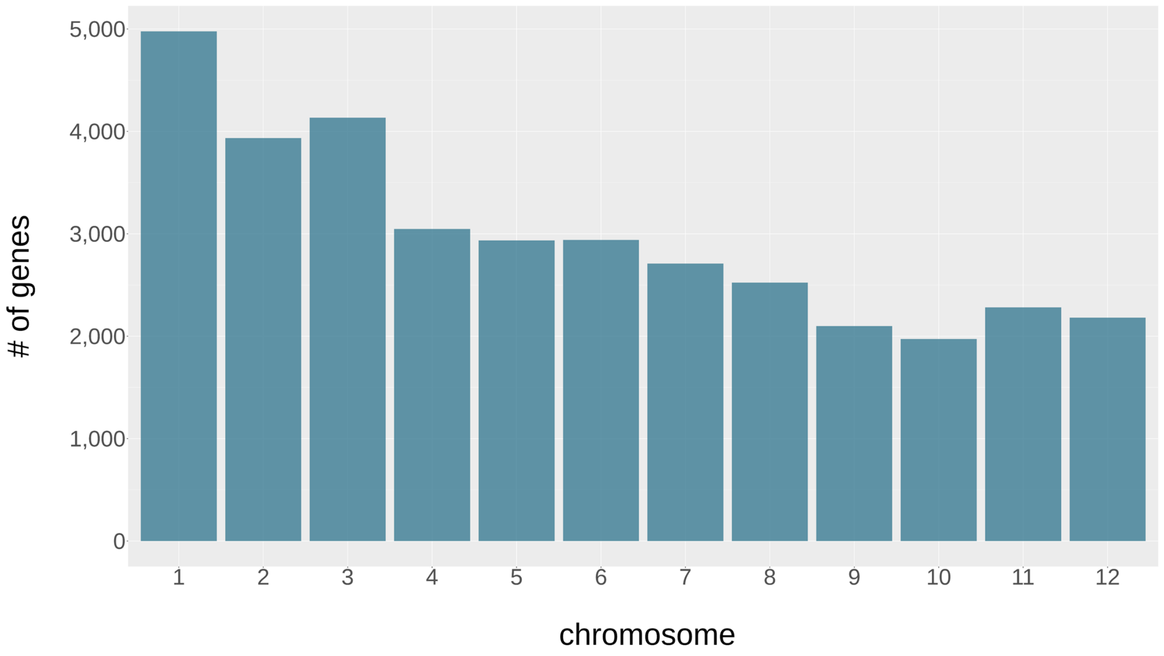

Figure S1.13: Total number of SNPs and genes for each chromosome of wild rice (*Oryza glumipatula*). (A) shows the number of SNPs per chromosome. (B) shows the number of genes per chromosome. For this plot, only the main chromosomes are included, excluding all unmapped contigs/scaffolds, random and mitochondrial chromosomes.
