# Supplementary material for: agReg-SNPdb-Plants: A Database of Regulatory SNPs for Agricultural Plant Species
Source: Biology (Basel). 2022 Apr 29;11(5):684. doi: 10.3390/biology11050684 (PMC9138521; doi:10.3390/biology11050684)
Supplement: Supplementary file 1 [file biology-11-00684-s001.zip › Suppl_FiguresS2_rSNPs_dist_to_TSS.pdf]

Supplementary Figures S2: Distribution of rSNPs around the TSS for each plant stored in agReg-SNPdb-Plants.

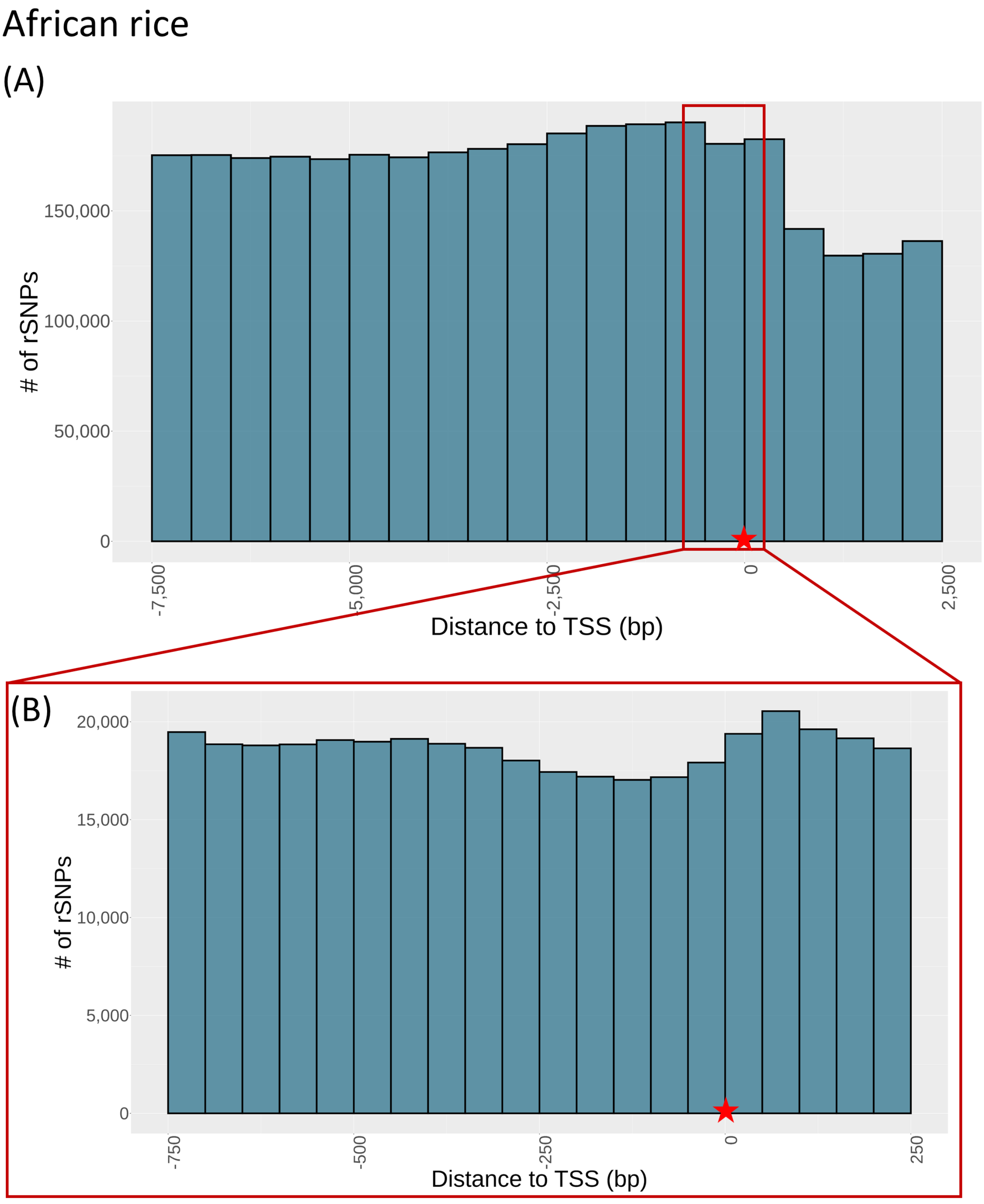

Figure S2.1: Distribution of rSNPs around the TSS of African rice (*Oryza glaberrima*). (A) shows counts for the entire region analyzed (-7.5 kb to +2.5 kb relative to the TSS) at 500-bp intervals. The magnification in (B) shows the proximal promoter region (-750 bp to +250 bp relative to the TSS) at 50-bp intervals. The red asterisk annotates the position of the TSS.

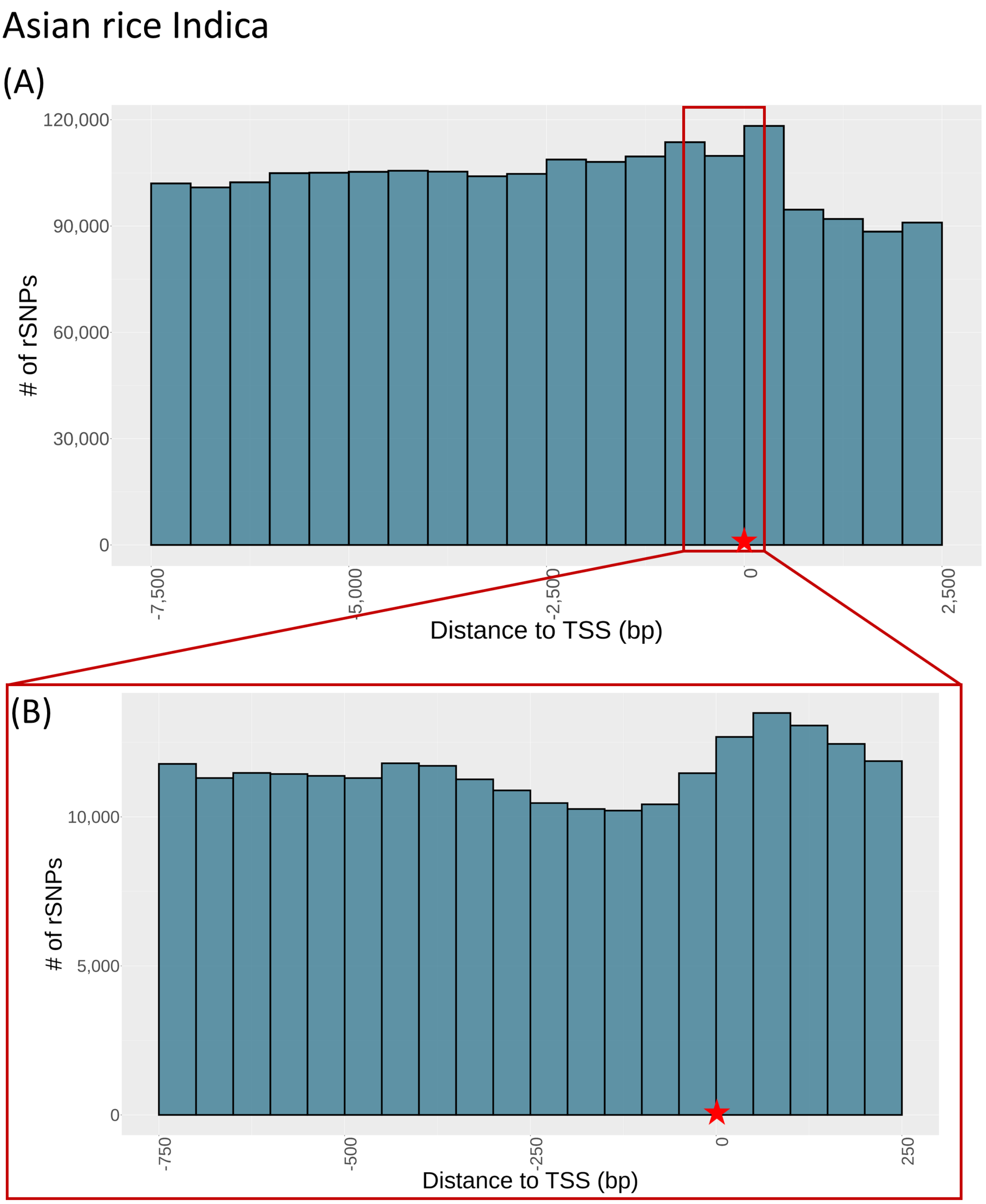

Figure S2.2: Distribution of rSNPs around the TSS of Asian rice Indica (*Oryza sativa* Indica). (A) shows counts for the entire region analyzed (-7.5 kb to +2.5 kb relative to the TSS) at 500-bp intervals. The magnification in (B) shows the proximal promoter region (-750 bp to +250 bp relative to the TSS) at 50-bp intervals. The red asterisk annotates the position of the TSS.

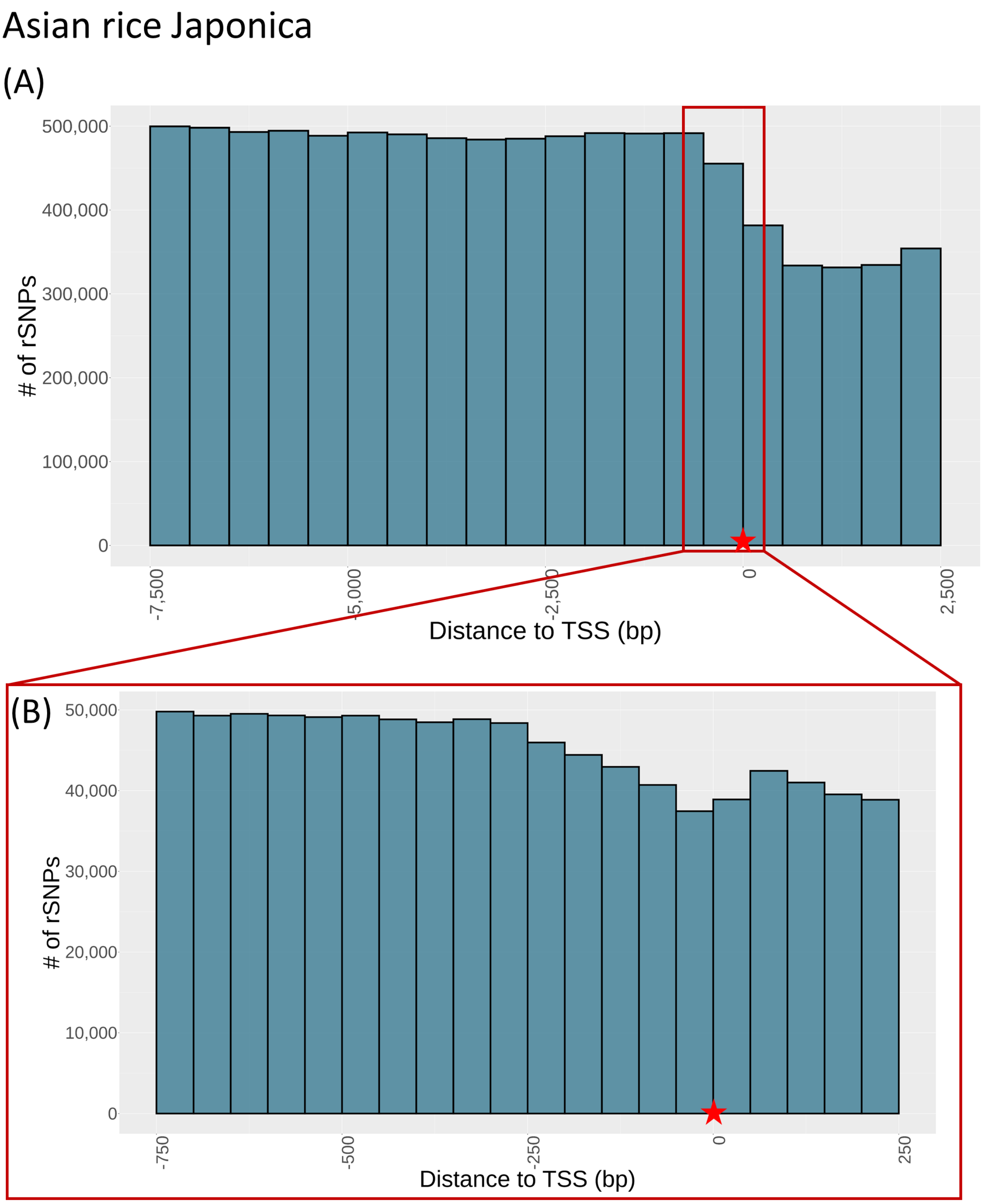

Figure S2.3: Distribution of rSNPs around the TSS of Asian rice Japonica (*Oryza sativa* Japonica). (A) shows counts for the entire region analyzed (-7.5 kb to +2.5 kb relative to the TSS) at 500-bp intervals. The magnification in (B) shows the proximal promoter region (-750 bp to +250 bp relative to the TSS) at 50-bp intervals. The red asterisk annotates the position of the TSS.

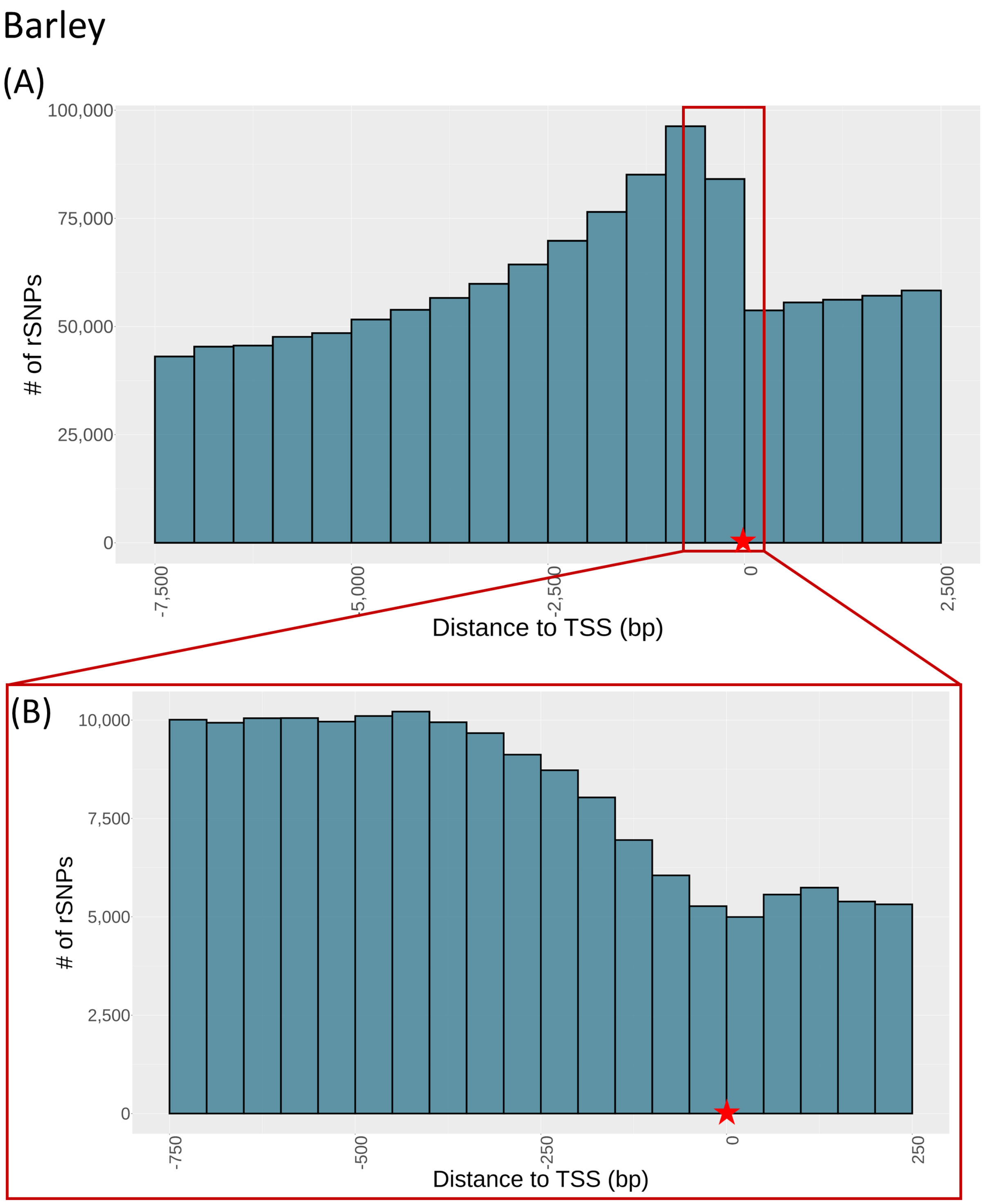

Figure S2.4: Distribution of rSNPs around the TSS of barley (*Hordeum vulgare*). (A) shows counts for the entire region analyzed (-7.5 kb to +2.5 kb relative to the TSS) at 500-bp intervals. The magnification in (B) shows the proximal promoter region (-750 bp to +250 bp relative to the TSS) at 50-bp intervals. The red asterisk annotates the position of the TSS.

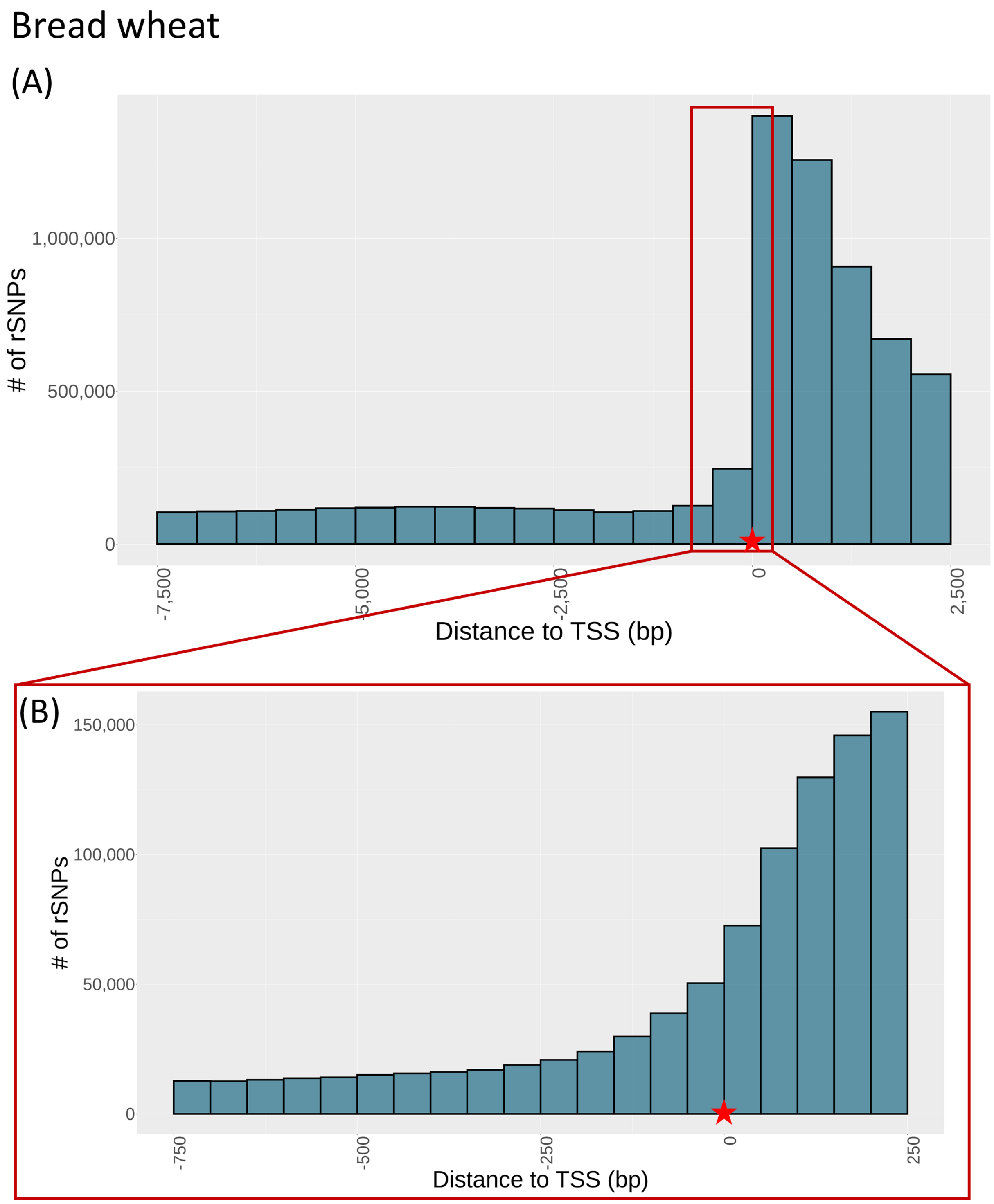

Figure S2.5: Distribution of rSNPs around the TSS of bread wheat (*Triticum aestivum*). (A) shows counts for the entire region analyzed (-7.5 kb to +2.5 kb relative to the TSS) at 500-bp intervals. The magnification in (B) shows the proximal promoter region (-750 bp to +250 bp relative to the TSS) at 50-bp intervals. The red asterisk annotates the position of the TSS.

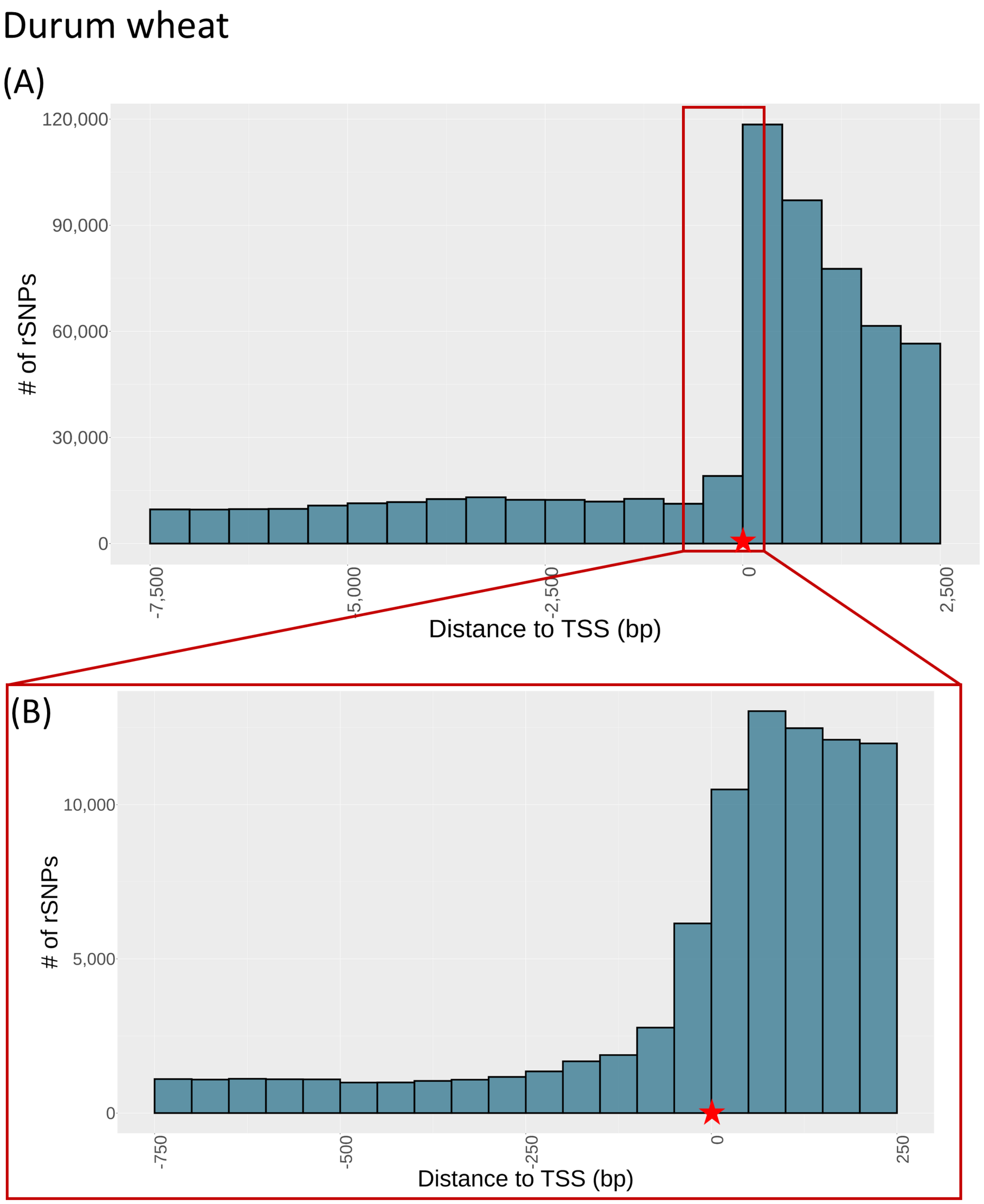

Figure S2.6: Distribution of rSNPs around the TSS of durum wheat (*Triticum turgidum*). (A) shows counts for the entire region analyzed (-7.5 kb to +2.5 kb relative to the TSS) at 500-bp intervals. The magnification in (B) shows the proximal promoter region (-750 bp to +250 bp relative to the TSS) at 50-bp intervals. The red asterisk annotates the position of the TSS.

Grape

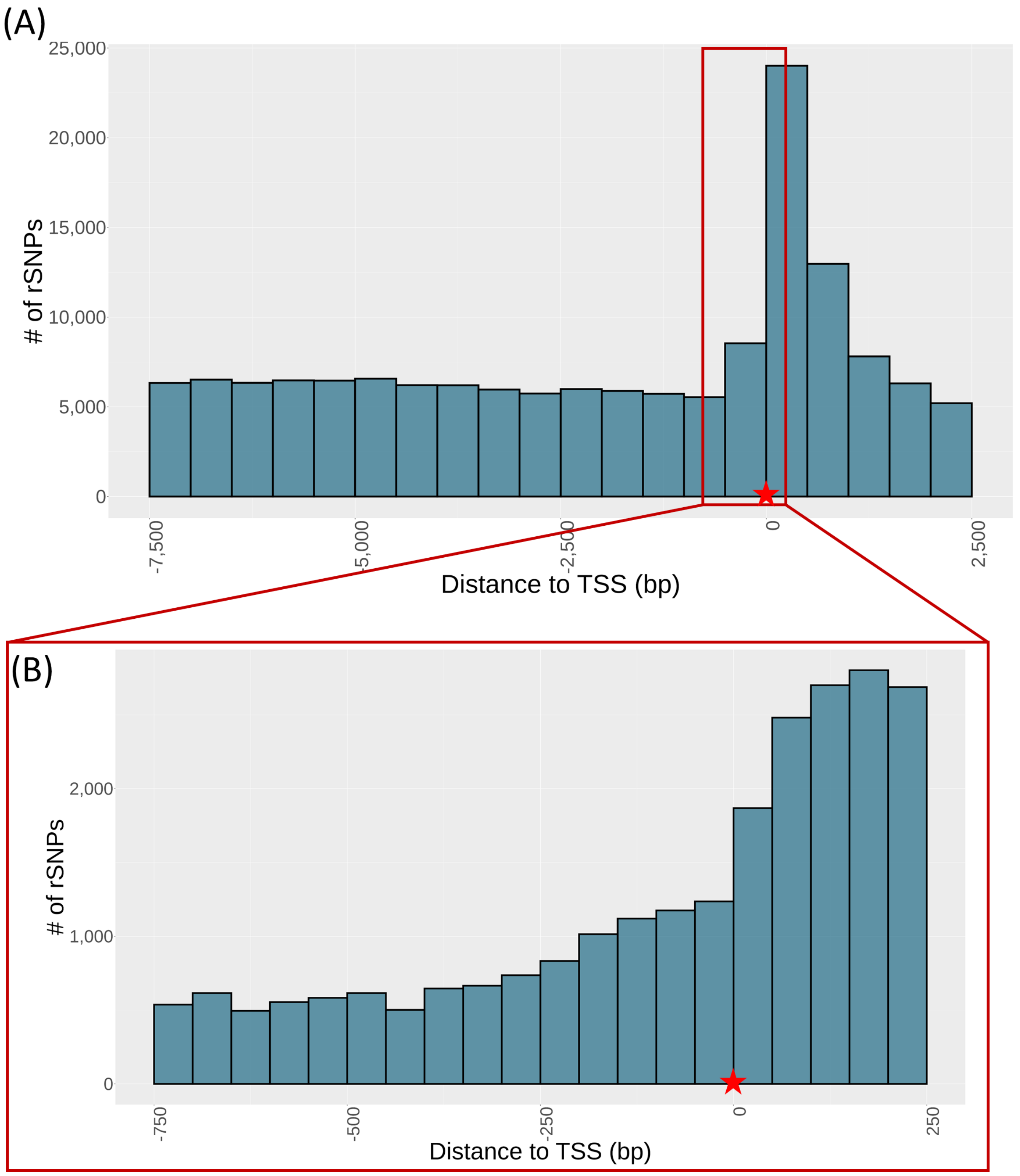

Figure S2.7: Distribution of rSNPs around the TSS of grape (*Vitis vinifera*). (A) shows counts for the entire region analyzed (-7.5 kb to +2.5 kb relative to the TSS) at 500-bp intervals. The magnification in (B) shows the proximal promoter region (-750 bp to +250 bp relative to the TSS) at 50-bp intervals. The red asterisk annotates the position of the TSS.

Maize

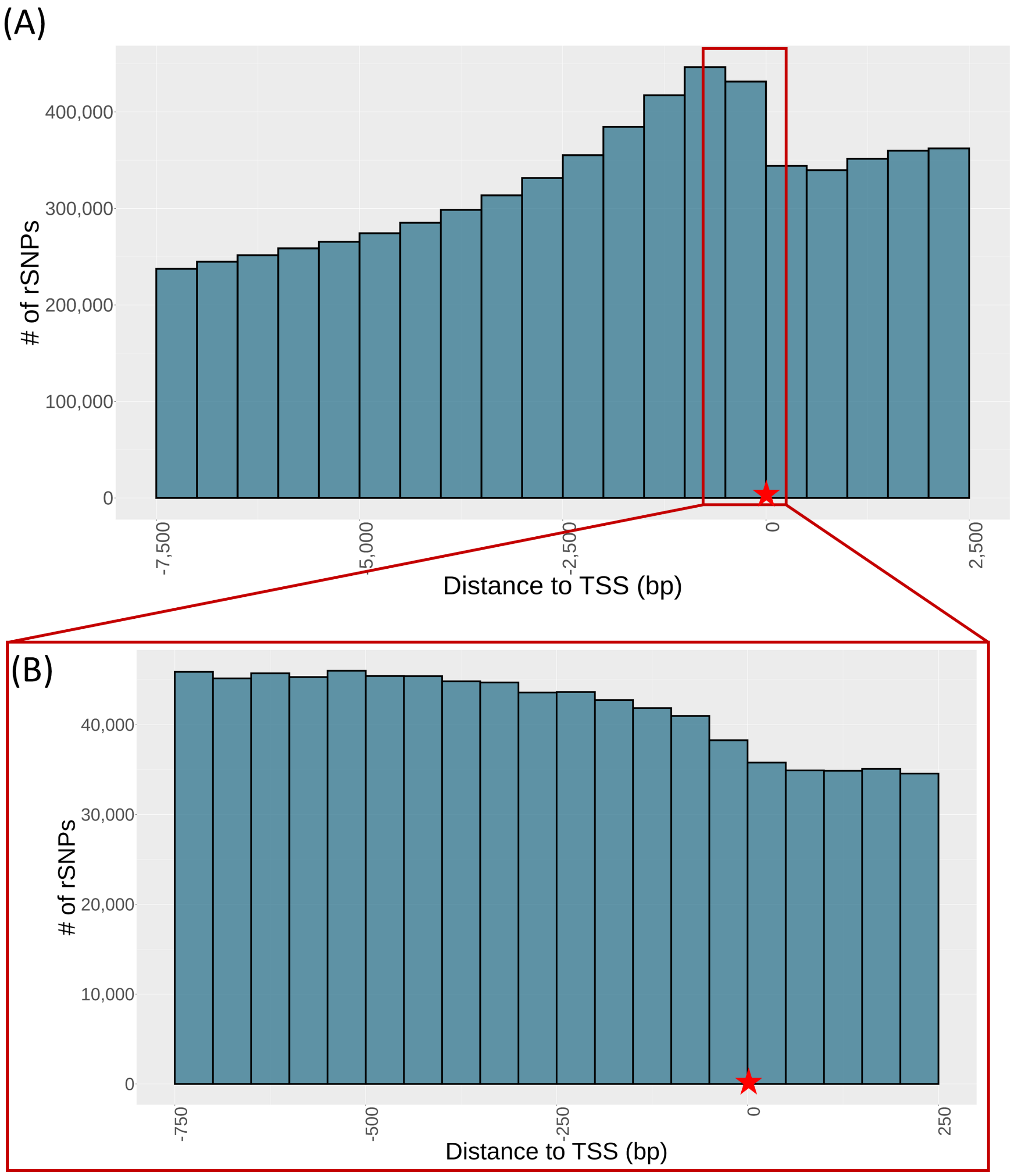

Figure S2.8: Distribution of rSNPs around the TSS of maize (*Zea mays*). (A) shows counts for the entire region analyzed (-7.5 kb to +2.5 kb relative to the TSS) at 500-bp intervals. The magnification in (B) shows the proximal promoter region (-750 bp to +250 bp relative to the TSS) at 50-bp intervals. The red asterisk annotates the position of the TSS.

Rapeseed

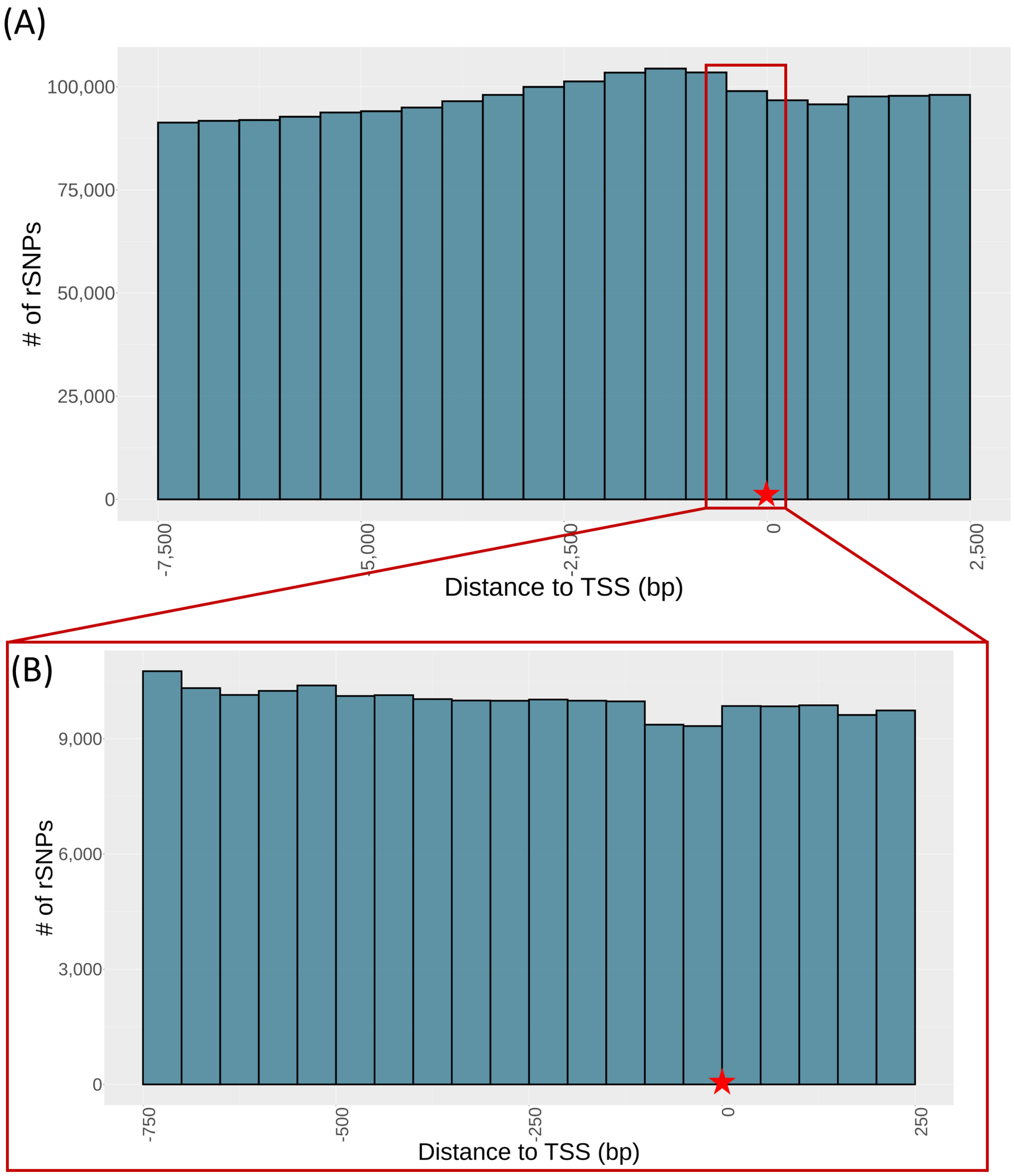

Figure S2.9: Distribution of rSNPs around the TSS of rapeseed (*Brassica napus*). (A) shows counts for the entire region analyzed (-7.5 kb to +2.5 kb relative to the TSS) at 500-bp intervals. The magnification in (B) shows the proximal promoter region (-750 bp to +250 bp relative to the TSS) at 50-bp intervals. The red asterisk annotates the position of the TSS.

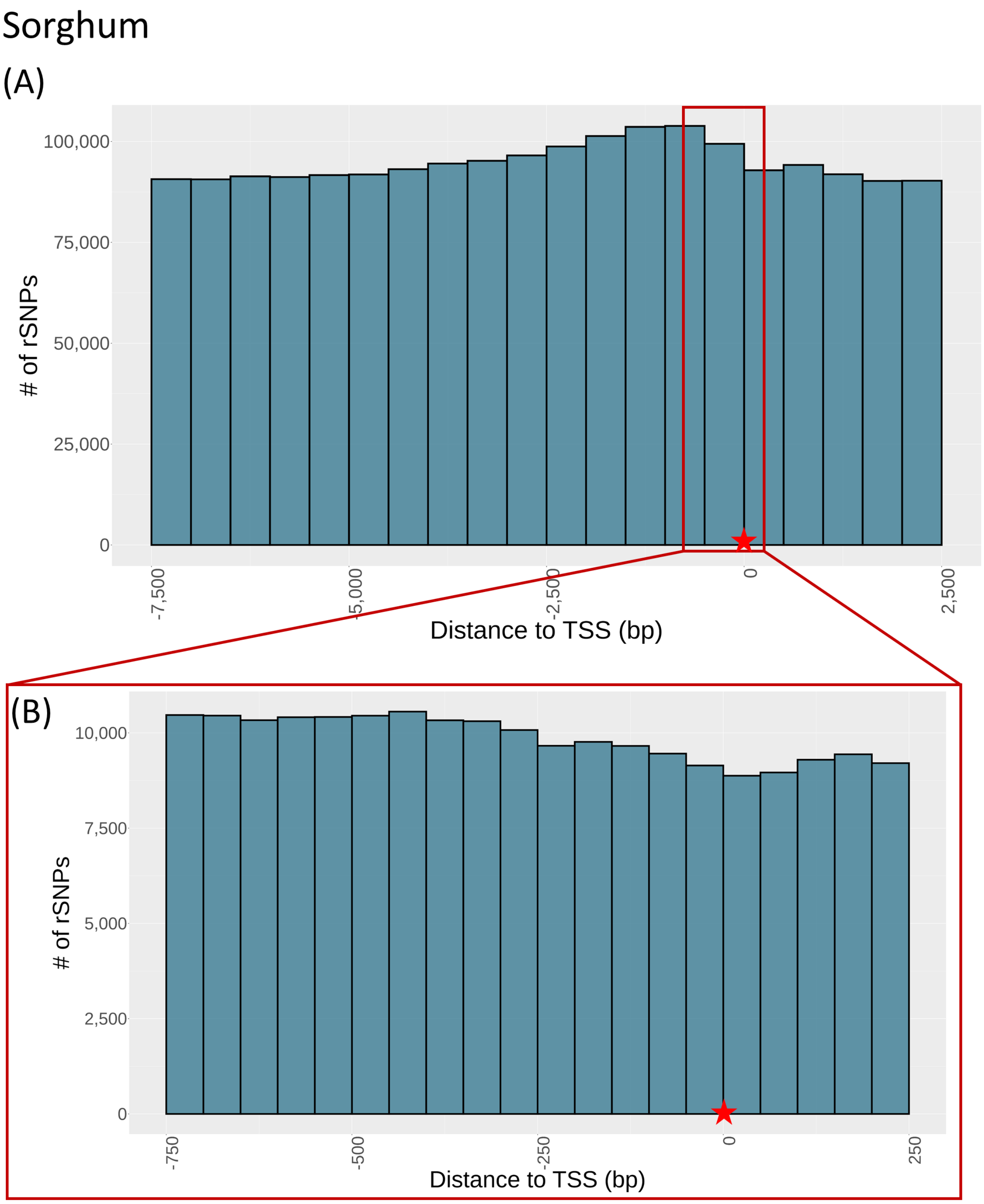

Figure S2.10: Distribution of rSNPs around the TSS of sorghum (*Sorghum bicolor*). (A) shows counts for the entire region analyzed (-7.5 kb to +2.5 kb relative to the TSS) at 500-bp intervals. The magnification in (B) shows the proximal promoter region (-750 bp to +250 bp relative to the TSS) at 50-bp intervals. The red asterisk annotates the position of the TSS.

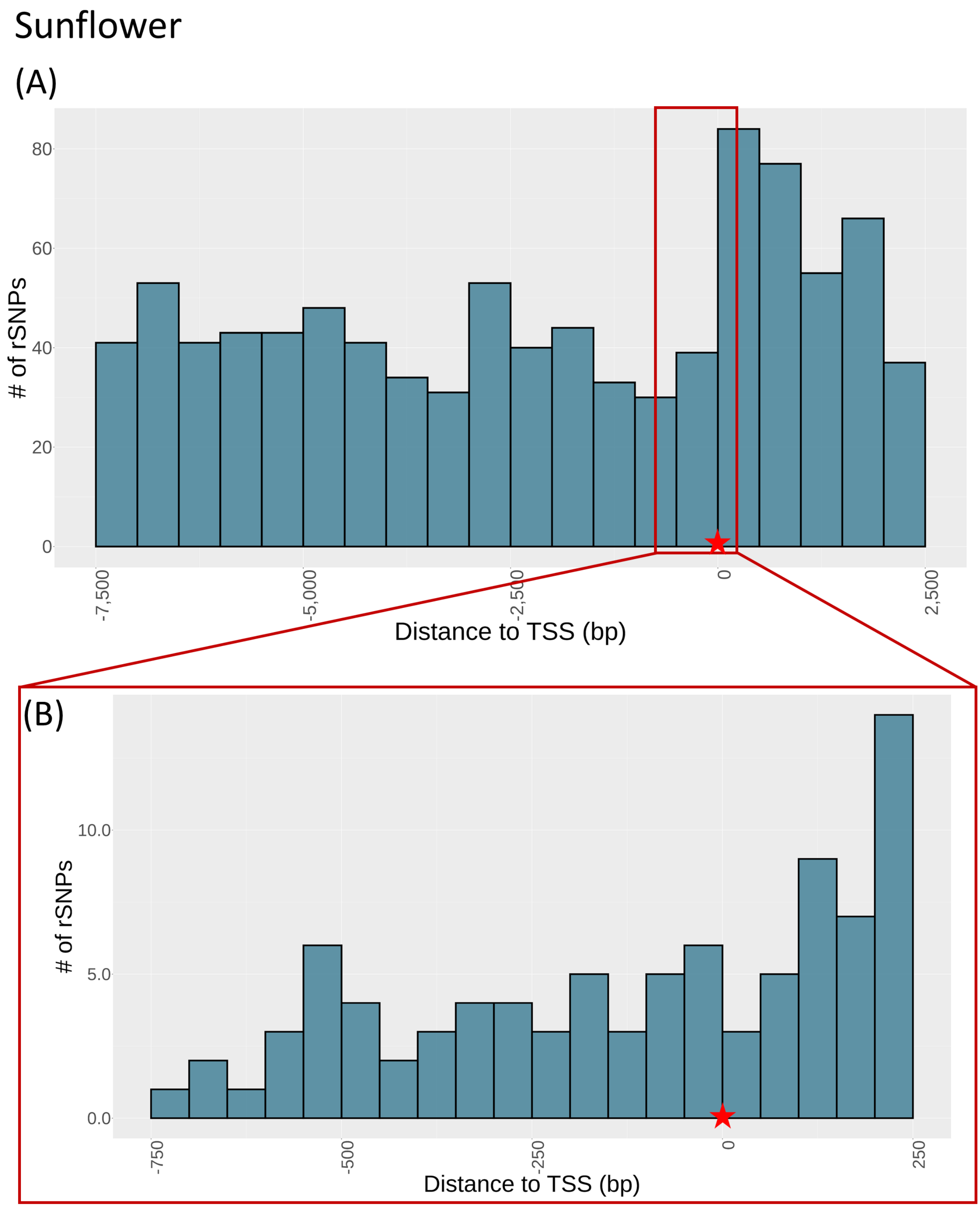

Figure S2.11: Distribution of rSNPs around the TSS of sunflower (*Helianthus annuus*). (A) shows counts for the entire region analyzed (-7.5 kb to +2.5 kb relative to the TSS) at 500-bp intervals. The magnification in (B) shows the proximal promoter region (-750 bp to +250 bp relative to the TSS) at 50-bp intervals. The red asterisk annotates the position of the TSS.

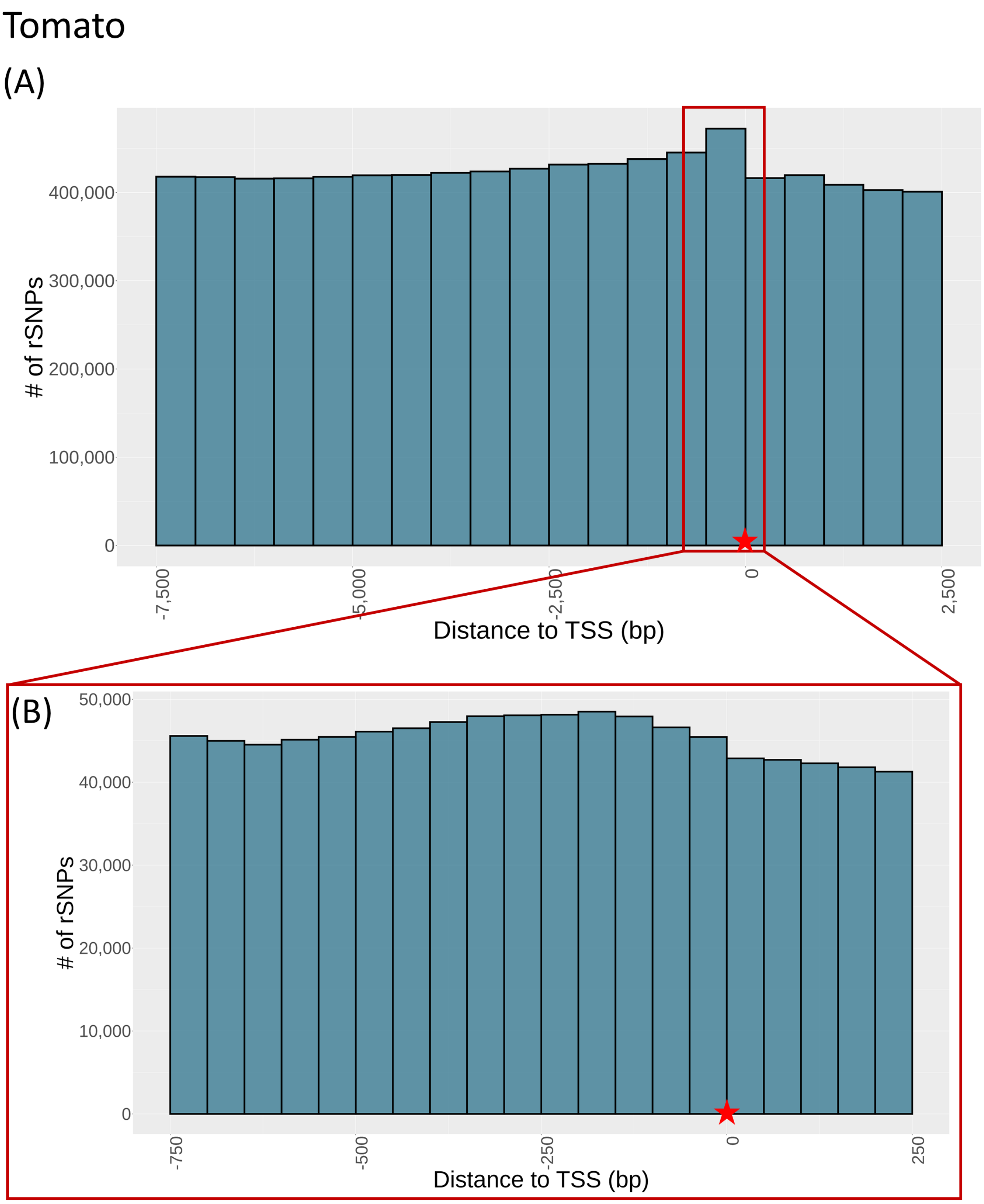

Figure S2.12: Distribution of rSNPs around the TSS of tomato (*Solanum lycopersicum*). (A) shows counts for the entire region analyzed (-7.5 kb to +2.5 kb relative to the TSS) at 500-bp intervals. The magnification in (B) shows the proximal promoter region (-750 bp to +250 bp relative to the TSS) at 50-bp intervals. The red asterisk annotates the position of the TSS.

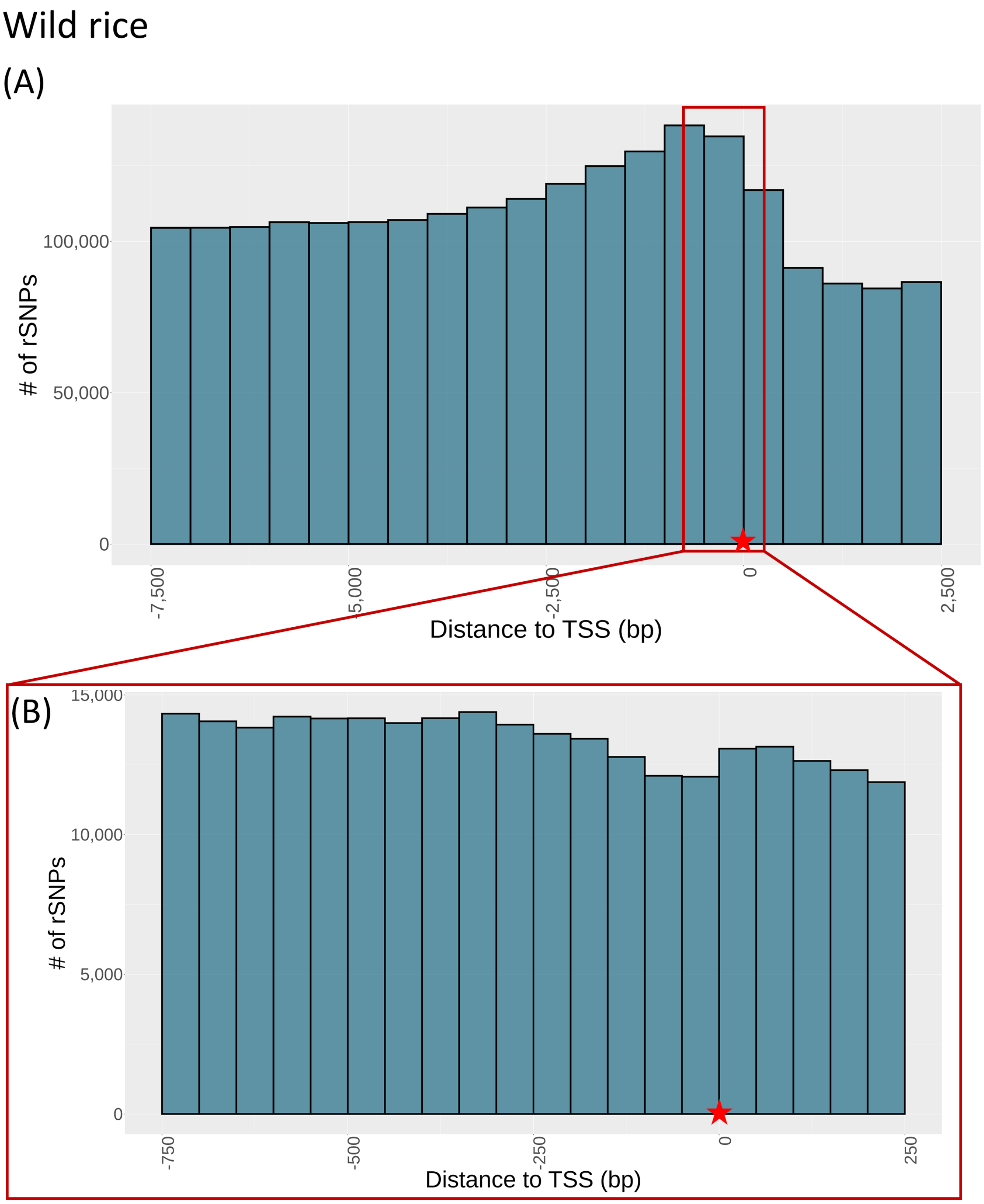

Figure S2.13: Distribution of rSNPs around the TSS of wild rice (*Oryza glumipatula*). (A) shows counts for the entire region analyzed (-7.5 kb to +2.5 kb relative to the TSS) at 500-bp intervals. The magnification in (B) shows the proximal promoter region (-750 bp to +250 bp relative to the TSS) at 50-bp intervals. The red asterisk annotates the position of the TSS.
