# Supplementary material for: agReg-SNPdb-Plants: A Database of Regulatory SNPs for Agricultural Plant Species
Source: Biology (Basel). 2022 Apr 29;11(5):684. doi: 10.3390/biology11050684 (PMC9138521; doi:10.3390/biology11050684)
Supplement: Supplementary file 1 [file biology-11-00684-s001.zip › Suppl_TableS1.pdf]

Supplementary Table S1: Textual evidences of different promoter definitions used by different studies for TFBS prediction or similar analyses. The table shows that previous studies are not clearly determined to one definition of a promoter region, but many different definitions exist in the literature ranging from -10 kb to +10 kb.

Study	promoter region	respective quotation
Klees et al. 2021 [1]	-7.5 kb → +2.5 kb	"For our analysis pipeline, we defined a relatively wide promoter region of 7.5 kb upstream to 2.5 kb downstream of the TSS."
Amlie-Wolf et al. 2018 [2]	-1 kb	"Promoter annotations were defined as 1000 bp upstream of the first exon in the transcript, either coding or in the UTR."
Guo et al. 2017 [3]	-2 kb → +1 kb	"Genome locations of the three types of regulatory elements were compared with the potential promoter region (from 2k upstream to 1k downstream of transcription start sites) of all Ensembl recorded genes on hg19 coordinate."
Ryan et al. 2014 [4]	1 kb	" [...] promoter (defined as being within 1 kb of a transcription start site (TSS) [...]"
Fu et al. 2014 [5]	-2.5 kb	"We focused on variants occurring in promoters (defined as -2.5 kb from transcription starting sites) [...]"
Riva et al. 2012 [6]	-5 kb → +200 bp	"To analyze SNPs in the gene's promoter, the user could instead use 5,000bp upstream of the transcript start and 200bp downstream of the transcript start as the region boundaries." (with the possibility to search for a wider region)
Kwon et al. 2012 [7]	-10 kb →+10 kb	"For human and mouse, 10,000 bp upstream and 10,000 bp downstream from the Ensembl-annotated TSS were searched for TFBS hits."
Coetzee et al. 2012 [8]	-1 kb → +100 bp	"The window parameter for promoters is -1000 to +100 bp from the TSS."
Ho et al 2005 [9]	-5 kb → +5 kb	"For all of the analyses presented in this study, we examined the promoter region encompassing 5000 bp upstream and 5000 bp downstream of the TSS, [...]"
Stepanova et al. 2005 [10]	-2 kb → +1 bp	"We used the 'classic' definition of a promoter as the gene area located between the positions -2000 to +1 according to the major transcription start site, [...]"

References

1. Klees, S.; Heinrich, F.; Schmitt, A.O.; Gültas, M. agReg-SNPdb: A Database of Regulatory SNPs for Agricultural Animal Species. *Biology* **2021**, *10*, 790.
2. Amlie-Wolf A., Tang M., Mlynarski E.E., Kuksa P.P., Valladares O., Katanic Z., Tsuang D., Brown C.D., Schellenberg G.D., Wang L.-S.. INFERNO: inferring the molecular mechanisms of noncoding genetic variants. *Nucleic acids research* **2018**, *46*, 8740–8753.
3. Guo L., Wang J.. rSNPBase 3.0: an updated database of SNP-related regulatory elements, element-gene pairs and SNP-based gene regulatory networks. *Nucleic acids research* **2017**, *46*, D1111–D1116.
4. Ryan, N.M.; Morris, S.W.; Porteous, D.J.; Taylor, M.S.; Evans, K.L. SuRFing the genomics wave: an R package for prioritising SNPs by functionality. *Genome medicine* **2014**, *6*, 79.
5. Fu Y., Liu Z., Lou S., Bedford J., Mu X.J., Yip K.Y., Khurana E., Gerstein M.. FunSeq2: a framework for prioritizing noncoding regulatory variants in cancer. *Genome Biology* **2014**, *15*, 480.
6. Riva, A. Large-scale computational identification of regulatory SNPs with rSNP-MAPPER. BMC genomics. BioMed Central, 2012, Vol. 13, p. S7.
7. Kwon, A.T.; Arenillas, D.J.; Hunt, R.W.; Wasserman, W.W. oPOSSUM-3: advanced analysis of regulatory motif over-representation across genes or ChIP-Seq datasets. *G3: Genes, Genomes, Genetics* **2012**, *2*, 987–1002.
8. Coetzee S.G., Rhie S.K., Berman B.P., Coetzee G.A., Noushmehr H.. FunciSNP: an R/bioconductor tool integrating functional non-coding data sets with genetic association studies to identify candidate regulatory SNPs. *Nucleic acids research* **2012**, *40*, e139–e139.
9. Ho Sui, S.J.; Mortimer, J.R.; Arenillas, D.J.; Brumm, J.; Walsh, C.J.; Kennedy, B.P.; Wasserman, W.W. oPOSSUM: identification of over-represented transcription factor binding sites in co-expressed genes. *Nucleic acids research* **2005**, *33*, 3154–3164.
10. Stepanova M., Tiazhelova T., Skoblov M., Baranova A. A comparative analysis of relative occurrence of transcription factor binding sites in vertebrate genomes and gene promoter areas. *Bioinformatics* **2005**, *21*, 1789–1796.
